# Integrative metagenomic, transcriptomic, and proteomic analysis reveal the microbiota-host interplay in early-stage lung adenocarcinoma among non-smokers

**DOI:** 10.1186/s12967-024-05485-0

**Published:** 2024-07-13

**Authors:** Yaohui Sun, Zhiming Gan, Xiaojin Wang, Jian Liu, Wei Zhong, Zhiyan Zhang, Jiebin Zuo, Hang Zhong, Xiuting Huang, Zhixiang Yan, Qingdong Cao

**Affiliations:** 1 Department of Thoracic Surgery and Lung Transplantation, The Fifth Affiliated Hospital of Sun Yat-Sen University, Zhuhai, 519000 Guangdong China; 2 Guangdong Provincial Key Laboratory of Biomedical Imaging and Guangdong Provincial Engineering Research Center of Molecular Imaging, The Fifth Affiliated Hospital of Sun Yat-Sen University, Zhuhai, 519000 Guangdong China; 3 Cardiovascular Disease Center, The Fifth Affiliated Hospital of Sun Yat-Sen University, Zhuhai, 519000 Guangdong China

**Keywords:** ES-LUAD, Intratumoral microbiome, Multi-omics, Correlation analysis, Non-invasive diagnosis

## Abstract

**Background:**

The incidence of early-stage lung adenocarcinoma (ES-LUAD) is steadily increasing among non-smokers. Previous research has identified dysbiosis in the gut microbiota of patients with lung cancer. However, the local microbial profile of non-smokers with ES-LUAD remains largely unknown. In this study, we systematically characterized the local microbial community and its associated features to enable early intervention.

**Methods:**

A prospective collection of ES-LUAD samples (46 cases) and their corresponding normal tissues adjacent to the tumor (41 cases), along with normal lung tissue samples adjacent to pulmonary bullae in patients with spontaneous pneumothorax (42 cases), were subjected to ultra-deep metagenomic sequencing, host transcriptomic sequencing, and proteomic sequencing. The obtained omics data were subjected to both individual and integrated analysis using Spearman correlation coefficients.

**Results:**

We concurrently detected the presence of bacteria, fungi, and viruses in the lung tissues. The microbial profile of ES-LUAD exhibited similarities to NAT but demonstrated significant differences from the healthy controls (HCs), characterized by an overall reduction in species diversity. Patients with ES-LUAD exhibited local microbial dysbiosis, suggesting the potential pathogenicity of certain microbial species. Through multi-omics correlations, intricate local crosstalk between the host and local microbial communities was observed. Additionally, we identified a significant positive correlation (rho > 0.6) between *Methyloversatilis discipulorum* and GOLM1 at both the transcriptional and protein levels using multi-omics data. This correlated axis may be associated with prognosis. Finally, a diagnostic model composed of six bacterial markers successfully achieved precise differentiation between patients with ES-LUAD and HCs.

**Conclusions:**

Our study depicts the microbial spectrum in patients with ES-LUAD and provides evidence of alterations in lung microbiota and their interplay with the host, enhancing comprehension of the pathogenic mechanisms that underlie ES-LUAD. The specific model incorporating lung microbiota can serve as a potential diagnostic tool for distinguishing between ES-LUAD and HCs.

**Supplementary Information:**

The online version contains supplementary material available at 10.1186/s12967-024-05485-0.

## Introduction

The escalating incidence of early-stage tumors has become a global concern [1], particularly with the increasing detection rates of early-stage lung adenocarcinoma (ES-LUAD) over the past decade. Smoking is recognized as a risk factor for lung cancer; however, a significant proportion of patients with lung cancer have never been exposed to cigarette smoke, particularly among the younger population [2, 3]. Aside from recognized risk factors such as genetic susceptibility, secondhand smoke, and occupational exposure, recent reports suggest a potential association between the etiology of lung cancer and kitchen fumes and microplastics. However, these factors alone cannot entirely account for the disease burden [4–6]. Although tumor development may involve various factors, including environmental influences, it remains unclear whether patients with ES-LUAD exhibit specific molecular characteristics.

Microbiomes are understood as the overlooked “invisible organ” of the human body, serving as a communication medium that links various organs [7]. The microbial composition of the human lung has received far less attention than the extensively discussed gut microbiota, primarily due to the historical perception of its “sterile” nature [8, 9]. The advancement of high-throughput sequencing technologies in recent years has progressively revealed a close association between local pulmonary ecological imbalances and various diseases [10]. However, as a regulator of the immune system, its impact on host health is significant and cannot be overlooked. Jin et al. established a germ-free mouse model and discovered that respiratory local microbiota can directly modulate the immune microenvironment, particularly γδ T cells. Consequently, this modulation stimulates inflammation and enhances the proliferative potential of tumor cells [11]. Recent studies have predominantly demonstrated the crosstalk between intestinal microbiota and lung tumorigenesis, indicating that the “gut–lung axis” may impact tumor initiation, progression, and response to treatments [12, 13]. Qian et al. identified gut microbiota dysbiosis in patients with non-small-cell lung cancer (NSCLC) through multi-omics analysis, revealing the contribution of the *Prevotella copri*-nervonic acid/all-trans-retinoic acid axis to the cancer phenotype in mice [14]. Tsay et al. provided evidence indicating the upregulation of the ERK and PI3K signaling pathways associated with the enrichment of the lower respiratory microbiota [15]. Although specific fluid-based models for early lung cancer diagnosis are continuously advancing, many established diagnostic models lack the requisite specificity and sensitivity, significantly limiting their potentiality as biomarkers [16–18]. Moreover, the heterogeneity of the lung microbiota among different cancer subtypes (adenocarcinoma vs. squamous cell carcinoma [SCC]) implies distinct microbial features [19]. Although a limited number of studies have revealed the microbial composition of lung tissue, they lack multi-omics evidence [18, 20, 21]. Current research predominantly relies on 16S rRNA sequencing, which presents challenges in providing an in-depth characterization of the inherently low biomass in the lung microbiota.

The microbial habitat better reflects its local microenvironment; however, due to the anatomical distance within the gut-lung axis, the direct interaction between local tumors and microbiota better represents the host’s internal homeostatic regulation. Hence, intraoperative lower respiratory tumor tissue samples, compared to feces, sputum, and bronchoalveolar lavage fluid (BALF), more directly reflect the microbiota–environment interaction with the host while minimizing environmental contamination. We observed that primary spontaneous pneumothorax (PSP), commonly prevalent among young adults, is typically instigated by congenital lung bullae. These bullae often stem from developmental anomalies in specific lung tissue regions during fetal stages but generally do not affect the surrounding normal lung tissue [22]. Hence, in patients undergoing surgical resection of bullae associated with spontaneous PSP, the adjacent normal lung tissue is considered clinically most akin to healthy lung samples.

The limitation in studying lung microbiota lies in the substantially lower biomass compared to the gut microbiome. Thus, our research employs ultra-deep metagenomics for comprehensive genomic and functional characterization [23]. The average tissue metagenomic sequencing depth per sample is 40 Gbp, ensuring adequate microbial coverage even in the presence of excess host genomic DNA. Furthermore, the NovaSeq 6000, for the transcriptome, and liquid chromatography-tandem mass spectrometry (LC–MS/MS), for the proteome, were integrated with the metagenome to complement single-omics analysis and reveal the dynamic changes in the ES-LUAD biological system. This prospective cohort study serves to identify the microbial spectrum in patients with ES-LUAD and partially elucidates the local tumor microenvironment’s interaction with the host.

## Materials and methods

### Patient recruitment and sample collection

A total of 42 HCs (adjacent normal lung tissue to PSP), and 46 ES-LUAD tumor tissues, and 41 paired normal tissues adjacent to the tumor (NATs) from patients who underwent thoracoscopic surgery were recruited from the Fifth Affiliated Hospital of Sun Yat-Sen University (Guangdong, China) from July 2021 to August 2022. Before enrollment, samples were gathered following the protocol sanctioned by the local ethics committee (No. K41-1, 2021), and written consent was obtained from each participant. Upon admission, interviews were conducted to gather demographic, medical history, lifestyle, and other characteristic information, followed by comprehensive evaluations through laboratory tests and imaging studies. All subjects were required to meet the following criteria: no respiratory infections, no history of smoking, and no use of antibiotics, steroids, or probiotic treatment within the past 3 months. All diagnosis and treatment procedures followed international guidelines [24, 25]. According to the 8th edition standards set by the International Association for the Study of Lung Cancer (IASLC) regarding lung cancer TNM staging, only patients with stage I and stage II were included. All patients were newly diagnosed with ES-LUAD or PSP on the basis of postoperative pathological examination. The sampling procedure was controlled within 20 min and strictly adhered to aseptic techniques, ensuring that the cancer-adjacent tissue was at least 2 cm away from the tumor. As a result, 305 lung tissue samples were collected and stored at − 80 °C until multi-omics sequencing. The comprehensive clinical characteristics of the enrolled subjects are summarized in Table 1. Ultimately, after excluding 12 samples because of transcriptome quality control failure, 293 samples were included for subsequent analysis.

**Table 1:** Clinical baseline features of sample data

Clinical features	Total(n = 129)			p value (HC vs LUAD)	p value (HC vs NAT)
	HC(n = 42)	LUAD(n = 46)	NAT(n = 41)		
Age (years)	31.8 ± 14.4	55.4 ± 11.2	55.2 ± 11.8	p < 0.0001	p < 0.0001
< 19	9(21.4%)	0(0.0%)	0(0.0%)		
19–45	24(57.1%)	8(17.4%)	8(19.5%)		
> 45	9(21.4%)	38(82.6%)	33(80.5%)		
Sex				p < 0.0001	p < 0.0001
Male	33(78.6%)	11(23.9%)	10(24.4%)		
Female	9(21.4%)	35(76.1%)	31(75.6%)		
T stage				N/A	N/A
Tis	N/A	7(15.2%)	6(14.6%)		
T1	N/A	32(69.6%)	28(68.3%)		
T2	N/A	7(15.2%)	7(17.1%)		
N stage				N/A	N/A
N0	N/A	42(91.3%)	37(90.2%)		
N1	N/A	4(8.7%)	4(9.8%)		
Clinical stage				N/A	N/A
I	N/A	41(89.1%)	36(87.8%)		
II	N/A	5(10.9%)	5(12.2%)		
Tumor infiltration				N/A	N/A
AIS	N/A	5(10.9%)	4(9.8%)		
MIA	N/A	16(34.8%)	13(31.7%)		
IA	N/A	25(54.3%)	24(58.5%)		
Location				p < 0.001	p < 0.001
Left upper lobe	25(59.5%)	13(28.3%)	11(26.8%)		
Left lower lobe	0(%)	9(19.6%)	8(19.5%)		
Right upper lobe	15(35.7%)	14(30.4%)	12(29.3%)		
Right middle lobe	0(%)	4(8.7%)	4(9.8%)		
Right lower lobe	2(4.8%)	6(13.0%)	6(14.6%)		
Nodules				N/A	N/A
Single	N/A	28(60.9%)	28(68.3%)		
Multiple	N/A	18(39.1%)	13(31.7%)		
Component				N/A	N/A
pGGN	N/A	17(37.0%)	14(34.1%)		
mGGN	N/A	13(28.3%)	13(31.7%)		
SN	N/A	16(34.8%)	14(34.1%)		

Data are presented as the mean ± SD or frequency (%)

P values calculated by Wilcoxon rank-sum test or Pearson’s Chi-square test

p < 0.05 considered statistically significant

AIS, Adenocarcinoma in situ; MIA, Minimally invasive adenocarcinoma; IA, Invasive adenocarcinoma; pGGN, Pure ground glass nodules; mGGN, Mixed ground glass nodules; SN, Solid nodule; N/A, not available

### Genomic DNA extraction, library preparation, and metagenomic sequencing

The samples were transported under cold chain conditions to Macro & Micro-Test Med-Tech Co., Ltd. (Beijing), commissioning them for the sequencing process. Briefly, 0.2 μg of DNA per sample was used for DNA library preparations. The NEBNext^®^ UltraTM DNA Library Prep Kit for Illumina (NEB, USA, Catalog #: E7370L) was employed to generate sequencing libraries in accordance with the manufacturer’s guidelines. Initially, genomic DNA was sonicated to achieve 350-bp fragments, which were then subjected to end-polishing, A-tailing, and ligation with full-length Illumina sequencing adapters, followed by PCR amplification. PCR products were purified using the AMPure XP system (Beckman Coulter, Brea, CA). The library quality was assessed using the Agilent 5400 system, and quantification was performed via qPCR (1.5 nM). Pools of qualified libraries, determined by their effective concentrations, were sequenced on the NovaSeq 6000 platform with PE150 (Illumina), resulting in a total sequencing data volume of 40 Gbp.

### Metagenomic classification and functional annotation, and analysis pipeline

The raw data underwent a series of operations using Fastp [26] software, including quality analysis, read filtering, trimming, adapter removal, polyG/polyX tail trimming, and UMI preprocessing. In an effort to eliminate low-quality reads and exclude those containing a specific proportion of N bases, human-derived sequences were removed using HISAT2 [27] software. Subsequently, Kraken2 [28] software, in conjunction with a microbial database, was employed to identify the species present in the sample. Bracken [29] was then used to classify the results obtained from Kraken2, enabling Bayesian re-estimation of abundance to determine the composition and abundance of species. The “Decontam” package [30] was employed to eliminate contaminant DNA sequences, thereby enhancing the sequencing quality. The clean reads after filtering are depicted in Additional file 1: Fig. S1A. To enhance clarity and mitigate the possibility of microbial presence in only a limited number of samples, microbes that were present in < 20% of the single group were excluded, leaving 2088 species, which constituted 8.0% of the initial quantity. For functional annotation, HUMAnN 3.0 [31] was used to accurately and efficiently profile the abundance of microbial functional genes and pathways matching UniRef90 IDs and functional databases within the metagenomic sequencing data (clean data).

The species accumulation curve and rarefaction curve were generated to assess the adequacy of the sample size and the reasonableness of the metagenomic sequencing data volume in the patients with ES-LUAD and HCs, respectively (Additional file 1: Fig. S1B, C). We annotated all sequenced species and showed that the intrapulmonary microbiota mainly consisted of bacteria, followed by fungi, while archaea and viruses accounted for only a small part of the total (Additional file 1: Fig. S1D). Alpha-diversity analysis and Permutational Multivariate Analysis of Variance (PERMANOVA) were performed using the R package “vegan.” Group-specific biomarkers were identified through linear discriminant analysis (LDA) scores obtained from the LDA effect size (LEfSe) analysis (LDA > 2.0 and p < 0.05).

### RNA extraction, RNA-sequencing, and transcriptomic analysis

Frozen lung tissue RNA sample preparations used total RNA as the input material. Sequencing libraries were created according to the manufacturer’s recommendations using the NEBNext Ultra RNA Library Prep Kit for Illumina (NEB, USA, Catalog #: E7530L). Purified double-stranded cDNA underwent end repair, A-tailing, and sequencing adapter ligation. AMPure XP beads were employed for isolating cDNA with a preferred length of 370–420 bp, which was subsequently amplified by PCR. The resulting PCR products were re-purified using AMPure XP beads to construct the library, ensuring an effective concentration greater than 2 nM. Pooled qualified libraries were sequenced on the NovaSeq 6000 platform with PE150 (Illumina), generating a sequencing data volume of 7 Gbp.

The data were initially subjected to quality control using Fastp software to eliminate low-quality reads and remove adapters. Subsequently, Bowtie2 was used to mitigate the impact of rRNA. Next, HISAT2 was used to align the sequencing reads with the human reference sequence (GRCh38.p12). Finally, transcriptional quantification at the transcript level was performed using featuresCounts software [32]. The clean reads after filtering are depicted in Additional file 1: Fig. S5A. Differential expression analysis was conducted using DESeq2 to identify differentially expressed genes (DEGs). Functional enrichment analysis was performed and visualized using the OmicShare tools online platform (https://www.omicshare.com). Weighted Gene Co-expression Network Analysis (WGCNA) of mRNAs was constructed using OECloud tools (https://cloud.oebiotech.com).

### Peptide extraction and LC–MS/MS analysis

The ES-LUAD and HC lung tissue samples (∼ 10 mg) were homogenized and lysed in lysis buffer (6 M Guanidinium hydrochloride (GdmCl), 10 mM tris(2-carboxyethyl)phosphine (TCEP), 40 mM 2-Chloroacetamide, and 100 mM Tris, at a pH of 8.5). The samples were then centrifuged at 16,000*g* for 15 min to remove tissue debris. The lysate was then collected, and the protein concentration was measured using a NanoDrop 2000. The crude protein extract was diluted at a ratio of 1:10 with dilution buffer (10% (v/v) acetonitrile (ACN), and 25 mM Tris, pH 8.5) containing 1 μg sequencing-grade trypsin (1/50, w/w), and it was then digested overnight at 37 °C. The digested sample was acidified to a final concentration of 0.375% trifluoroacetic acid (TFA), and debris was removed after centrifugation at 16,000*g* for 15 min. Finally, the peptides were desalted on StageTips assembled using an Empore C18 disk, dried using a SpeedVac centrifuge at 45 °C, suspended in 2% ACN and 0.1% formic acid (FA), and loaded on Evosep tips.

The ES-LUAD and HC lung tissue samples were measured using an EvosepOne system coupled with Orbitrap Fusion Lumos Tribrid mass spectrometer. Chromatographic analysis was performed on a 15 cm × 150 μm capillary column packed with 1.9 μm C18 porous beads. Solvent A was 0.1% (v/v) FA in water, and solvent B was 0.1% (v/v) FA in ACN. Peptides were loaded onto the Evotip and eluted using a standard gradient at a rate of 30 samples per day, after which they were introduced into the mass spectrometer via a spray voltage of 2200 V. MS data were acquired using the data-dependent acquisition (DDA) mode, and for fragmentation, the higher energy collision dissociation (HCD) mode was used.

The proteomic raw data were processed using Proteome Discoverer 2.5 software [33] (PD2.5, Thermo Scientific) with the Sequest HT search engine, which allowed for peptide identification against the reference human database in UniProt (2017.06). The minimum peptide length was set to 6 amino acids, and enzyme specificity was configured to allow for three missed trypsin cleavage sites. The precursor and fragment mass tolerance were maintained at 10 ppm and 0.02 Da. Dynamic modifications were configured to include the acetylation of the protein N-terminus, the carbamidomethylation of Cys, the oxidation of Met, the deamidation of Asn and Gln, and the deamidation of Pyro-glu from Gln; there was a maximum allowance of three modifications. The False Discovery Rate (FDR) for both protein and peptide levels was set to 0.01 to ensure a high confidence level for peptide spectrum matching. The stability and repeatability of sample detection were assessed using a correlation heatmap (Additional file 1: Fig. S7A). Differentially expressed proteins (DEPs) were identified by Wilcoxon rank-sum test, and functional enrichment analysis was performed on the DEPs.

### Establishing random forest models to identify biomarkers

We explored the predictive and diagnostic value of multi-omics biomarkers in ES-LUAD using the random forest model (R package: randomForest [34]). Initially, individual omics-based random forest models were constructed using machine learning methods. These single-omic models were then integrated to explore their combined application value. Variables in all models were prioritized on the basis of their contribution scores, and an optimal feature count was selected following the principle of minimal cross-validation error. The model’s performance was validated by employing tenfold cross-validation to determine the best biomarkers through averaged validation results. Finally, the efficacy of the models was tested in an independent test set (splitting into a ratio of 75% and 25% for training and validation sets), and receiver operating characteristic curves (ROC) [35] were plotted.

### Validation and prognostic analysis based on public databases

We further validated our transcriptome data results using a public dataset. Gene Expression Profiling Interactive Analysis 2 [36] (GEPIA2) (http://gepia2.cancer-pku.cn/) incorporates transcriptome expression data from 9,736 tumors and 8,587 normal samples sourced from The Cancer Genome Atlas (TCGA) and Genotype-Tissue Expression (GTEx) projects, allowing for online visualization of results. Initially, we validated the DEGs against the database’s results (|log2 fold change|> 1; p < 0.05). We further assessed the correlation between DEGs and overall survival (OS) and disease-free survival (DFS) (Mantel–Cox test), and visually represented the correlation through survival maps and Kaplan–Meier curves to supplement prognostic data.

### Integrating multi-omics analysis

Spearman correlation was employed to explore the associations between multi-omics. Correlation pairs were assessed using the correlation coefficient values. The software Cytoscape [37] (version 3.9.1) was used to visualize the correlation networks.

### Statistical analysis

Unless otherwise specified, all analysis were performed using the software R (version: 4.1.2). Statistical analysis of clinical data was conducted using SPSS 26.0, with count data presented as frequencies and percentages (%). Two-group continuous variables (measured data) are presented as the mean ± standard deviation (X ± S). Comparisons were conducted for normally distributed and homoscedastic metrics between groups using the independent samples t-test (Student’s t-test), while categorical variables are evaluated using the Chi-square test or Fisher’s exact test. The comparison of non-normally distributed continuous variables between the two groups was conducted via two-sided Wilcoxon rank-sum tests, and the Kruskal–Wallis test was used for multiple groups. Results with a p-value < 0.05 were deemed statistically significant.

## Results

### Participant information

**Fig. 1:**
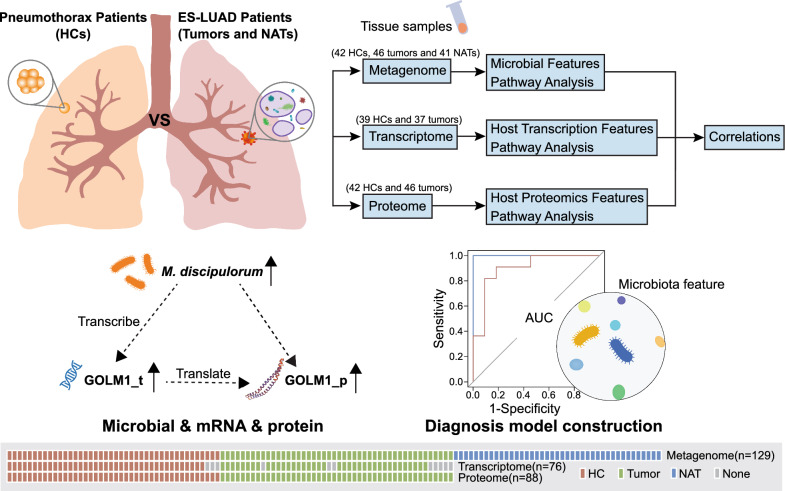
Overview of the study design and the number of samples for the metagenome, transcriptome, and proteome

All samples were collected from Han Chinese individuals residing in the southern region of China who shared similar dietary habits. Following a rigorous screening process that excluded any unsuitable samples, 129 tissue samples were incorporated into the study, with 41 paired samples originating from the same individuals. It was ensured that a minimum of three replicates were retained for each sample to facilitate multi-omics sequencing. Potential factors capable of influencing the composition of lung microbiota, such as smoking, hormone nebulization, and antibiotic use, were systematically excluded from the analysis. The study design is visually depicted in Fig. 1A. However, the demographic data highlighted significant distinctions among the groups in terms of age, sex, and lesion location (Table 1), which are primarily influenced by the epidemiological characteristics of ES-LUAD and PSP. Based on ultra-deep metagenome shotgun sequencing, a total of 129 samples generated 4848.2 Gbp of high-quality data, encompassing 140 million microbial reads.

### Microbial diversity and taxonomic composition in ES-LUAD

**Fig. 2:**
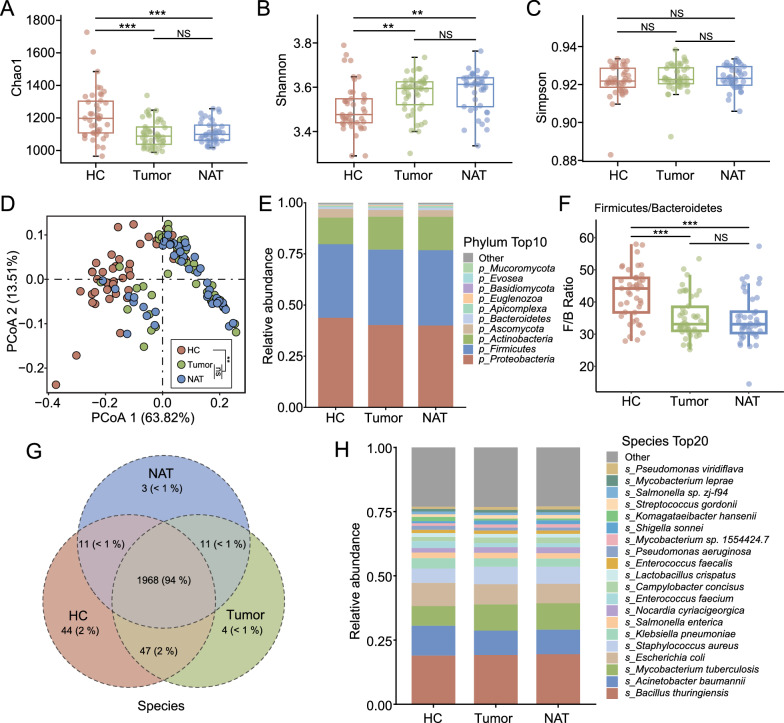
Characterization of the microbial diversity and taxonomic composition in patients with ES-LUAD and HCs. **A**–**C** Comparison of alpha diversity (Chao1/Shannon/Simpson index) at the species level in lung tissue of patients with ES-LUAD and HCs. **D** Comparison of beta diversity (Bray–Curtis distance) at the species level in the lung tissue of patients with ES-LUAD and HCs. **E** The average distribution of tumor, NAT, and HC groups in the phylum. **F** The ratio of Firmicutes and Bacteroidetes. **G** Venn diagram depicting shared and unique taxa for the tumor, NAT, and HC groups at the species level. **H** The average distribution of microbial species in the tumor, NAT, and HC groups. Box plots show the median ± quartiles, and the whiskers extend from the hinge to the largest or smallest value no further than 1.5-fold of the inter-quartile range. ns: Not significant, **p < 0.01, ***p < 0.001 as determined by Wilcoxon rank-sum test

Initial evaluation of the species diversity of the tumor, NAT, and HC samples revealed significant differences between them. Compared to the HCs, the lung microbiota of patients with ES-LUAD showed a notable reduction in the Chao1 index (p < 0.001), coupled with a pronounced elevation in the Shannon index (p < 0.001) (Fig. 2A, B). However, no significant variation was observed in the Simpson index across the three cohorts (Fig. 2C).

Further analysis of the intrapulmonary microbiota through beta diversity comparison using PERMANOVA and principal coordinate analysis (PCoA) with the Bray–Curtis distance metric confirmed a marked divergence in the bacterial community structure between patients with ES-LUAD and HCs (PERMANOVA test, p < 0.01) (Fig. 2D). These observations underscore the dysbiosis of intrapulmonary microbiota in patients with ES-LUAD.

At the taxonomic level, an average of three bacterial phyla, including Proteobacteria, Firmicutes, and Actinobacteria, accounted for more than 90% of the total microbial abundance. Notably, patients with ES-LUAD exhibited a diminished presence of *Proteobacteria* and an increased presence of *Actinobacteria* relative to HCs (Fig. 2E). A significant decrease in the *Firmicutes* to *Bacteroidetes* ratio was observed in patients with ES-LUAD (p < 0.001, Fig. 2F), which is indicative of a shift towards a less healthy state; this could potentially be linked to heightened pathogenicity [38, 39]. At the genus and species levels, a total of 1029 and 2088 classified taxa were identified, respectively. The predominant microbial communities across HC, tumor, and NAT tissues were remarkably similar (Fig. 2G, Additional File 1: Fig. S2A–C). The major bacterial genera across all tissue samples were *Bacillus*, *Acinetobacter*, *Mycobacterium*, and *Escherichia*, collectively comprising approximately half of the total abundance (Additional File 1: Fig. S2B). At the species level, *Bacillus thuringiensis*, *Acinetobacter baumannii*, *Mycobacterium tuberculosis*, and *Escherichia coli* were the most abundant taxa in both patients with ES-LUAD and HCs (Fig. 2H). The species composition of the corresponding single sample is shown in Additional File 1: Fig. S2A–C. Collectively, these data suggest that the microbial profiles of individuals with ES-LUAD showed similar microbial compositions.

### Taxonomic alterations of microbiota in patients with ES-LUAD

**Fig. 3:**
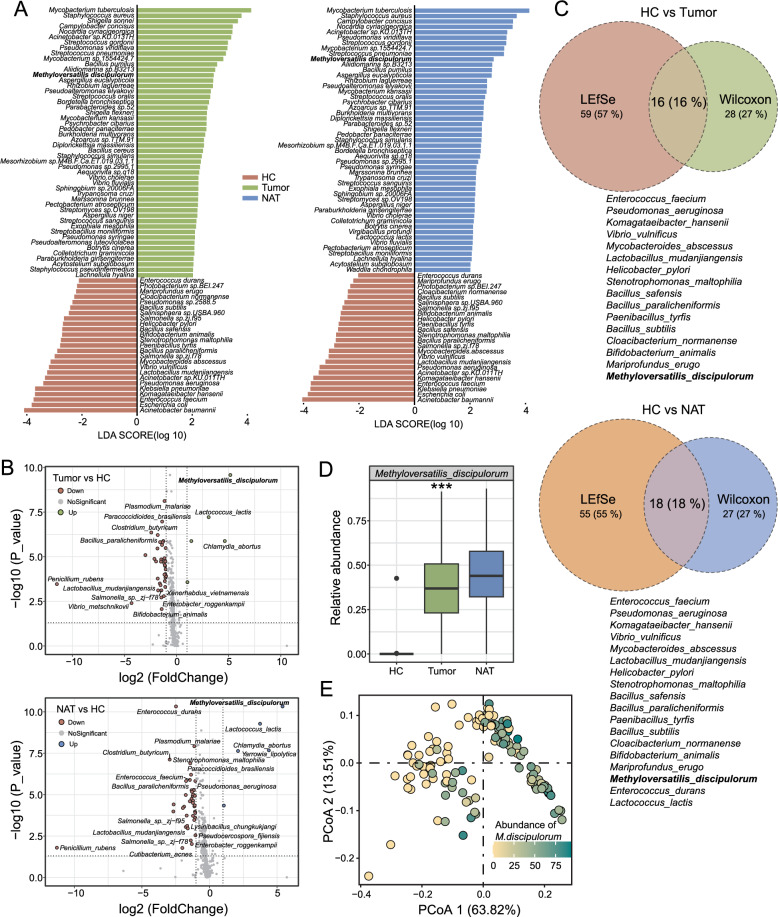
Intrapulmonary microbiota signatures of patients with ES-LUAD. **A** LEfSe analysis of differentially abundant intrapulmonary microbiota among tumor, NAT and HC groups. **B** Volcano diagram shows the different species among tumor, NAT and HC groups. **C** Venn diagram depicting shared and unique species for LEfSe analysis and Wilcoxon rank-sum test. **D** Boxplot showed representative microbial (*M. discipulorum*) that significantly changed among three groups, ***p < 0.001 as determined by Kruskal–Wallis test. **E** PCoA similar to Fig. 2D, with samples colored according to the abundance of *M. discipulorum*

To ensure the identification of active bacterial taxa, we meticulously curated our dataset by excluding those with less than 50 reads per sample. This stringent criterion led to the retention of 398 species for further analysis. Utilizing LEfSe, we discerned robust taxonomic signatures distinguishing HCs from ES-LUAD/NAT groups (Fig. 3A). A total of 75 differentially abundant species were identified between tumor and HC groups, and 73 species between NAT and HC groups (LDA score > 2.0 and p < 0.05). Additionally, the Wilcoxon rank-sum test identified 44 differentially abundant species in the ES-LUAD versus HC comparison and 45 in the NAT versus HC comparison (|log2FC|> 1, Fig. 3B). By integrating these two methodologies, we identified 16 distinct species in the HC and ES-LUAD groups and 18 unique species in the HC and ES-LUAD groups, all of which are considered to possess high confidence (Fig. 3C). Among these, 16 features corresponded to the same species, with 15 showing reduced abundance in patients with ES-LUAD (Additional file 1: Fig. S3). A subset of these enriched microbial communities in HCs included common gut bacteria, such as *Enterococcus faecium* and *Helicobacter pylori*, which may be associated with oral-nasal inhalation or microbial exchange. Notably, a heightened abundance of *M. discipulorum* was observed in the lung tissue of patients with ES-LUAD, contrasting with its near absence in HCs (LDA = 2.78, Fig. 3D, E). These signature microbial abundances were similar in the tumor and NAT samples, suggesting a similarity in their sources due to local microbial flow within the same individual [40].

Correlation analysis between intrapulmonary microbiota and critical clinical features further showed no significant association between the degree of tumor infiltration (p > 0.05, Additional file 1: Fig. S4A, D) and the diversity composition of the microbiota. Similar findings were observed in the context of the tumor’s solid component (p > 0.05, Additional file 1: Fig. S4B, E) and the presence of multiple primary nodules (p > 0.05, Additional file 1: Fig. S4C, F). While these clinical features may impact the prognosis in ES-LUAD, our data suggest that they are not significantly influenced by the pulmonary microbiota.

### Functional alteration of the lung tissue metagenome in ES-LUAD

**Fig. 4:**
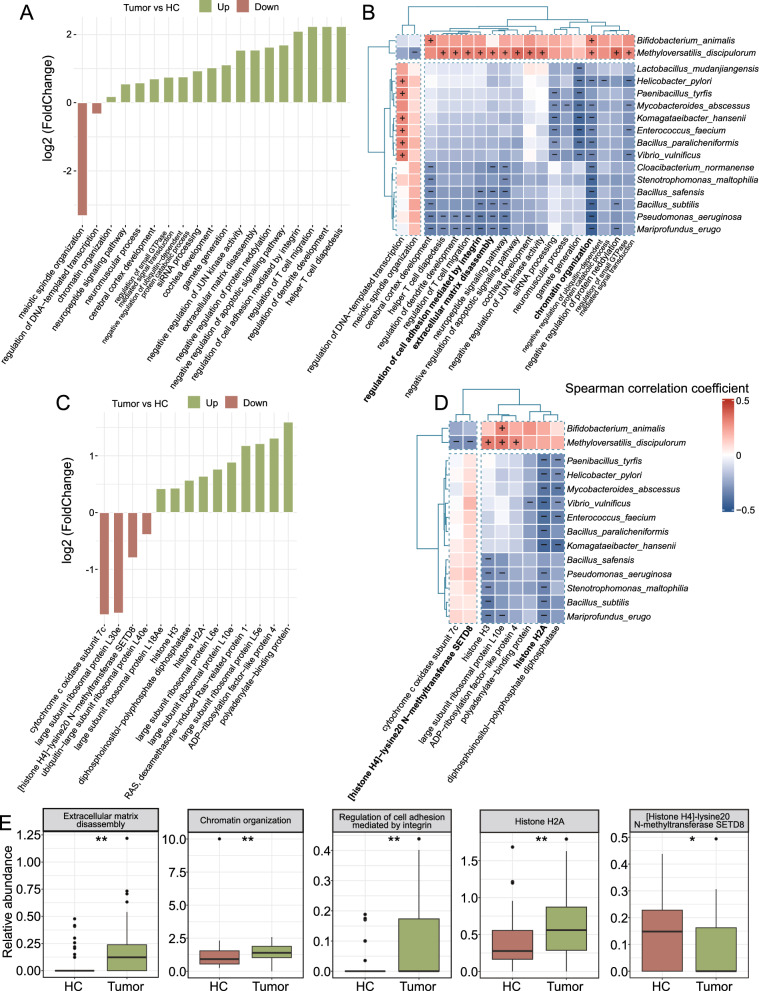
Microbiota-mediated functional gene alterations in patients with ES-LUAD. **A**, **C** The Bar plots show the significant GOs (**A**) and KO genes (**C**) between patients with ES-LUAD and HCs. (Wilcoxon rank-sum test, |log2FC|> 0.25, p < 0.05). **B**, **D** Correlation heatmap of microbial functions and signature microbial taxa. Correlations with |rho|> 0.3 and p < 0.05 were visualized. **E** Boxplot showing representative functional genes that significantly changed between patients with ES-LUAD and HCs. *p < 0.05, **p < 0.01 as determined by Wilcoxon rank-sum test

To explore the microbial biological pathway alterations in ES-LUAD, microbial genes from metagenomic analysis were annotated to the KEGG Orthology (KO) and Gene Ontology (GO) databases. A total of 197 KO genes and 2960 GO terms were identified after filtering for subsequent analysis. Compared to HCs, GO biological process analysis revealed significant enrichment in pathways such as regulation of cell adhesion mediated by integrins, chromatin organization, and extracellular matrix disassembly in ES-LUAD, while the processes of regulation of DNA-templated transcription and meiotic spindle organization were markedly suppressed (Fig. 4A, E). Correlation analysis revealed complex associations between signature microbial communities and pathways, while *M. discipulorum* potentially exhibited significant positive regulatory relationships (Fig. 4B). Additionally, 14 KEGG functional genes showed significant differences between tumor and HC samples, which were predominantly characterized by the upregulation of large subunit ribosomal proteins (RPs) such as L10e, RP-L6e, RP-L5e, and RP-L18Ae in the tumor samples, together with the depletion of RP-L40e and RP-L30e. This supports the notion that ribosomal activity is a pivotal molecular event in ES-LUAD [39]. Epigenetic changes in histones are known to modulate transcription at various gene loci, influencing tumor progression, metastasis, and drug resistance in NSCLC [40]. Our dataset showed that histone H2A and histone H3 were upregulated in the ES-LUAD group, which was accompanied by the downregulation of [histone H4]-lysine20 N-methyltransferase SETD8 (Fig. 4C, E). Correlation analysis suggested a significant negative correlation between signature microbial communities and histone H2A (Fig. 4D). Additionally, we found indications suggesting that *M. discipulorum* may be involved in the modification of histone H4 by the N-methyltransferase SETD8 (Fig. 4D). Taken together, these findings suggest that the functional alterations of microbiota play a specific role in the etiology of ES-LUAD.

### Associations between ES-LUAD-linked microbiota and host mRNAs

**Fig. 5:**
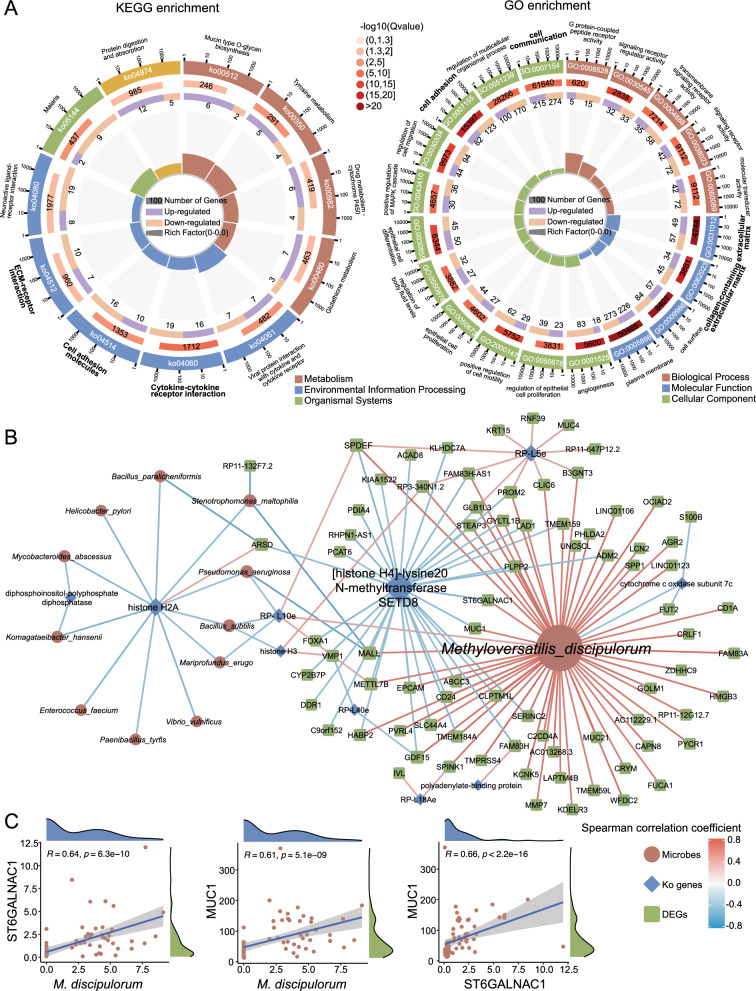
Host transcriptome alterations and their association with intrapulmonary microbiota. **A** KEGG and GO pathway enrichment analysis of gene sets with significant transcriptional differences between tumor and HC tissues. **B** Network diagram depicting correlations among signature microbial, microbial-related KO genes, and DEGs (p < 0.05). Microbes, KO genes, and DEGs are represented by orange circles, blue diamonds, and green squares respectively. The size of the shapes represents the number of nodes. The color scale represents the Spearman correlation coefficient. Only correlation pairs containing KO genes with |rho|> 0.35 and p < 0.05 were visualized, while correlation pairs containing microbes with |rho|> 0.6 and p < 0.05 were visualized. **C** Scatter plot representing the correlation between *M. discipulorum*, ST6GALNAC1, and MUC1 at the transcription level

To further identify dynamic interactions among signature microbiota, KO genes, and host characteristics, we performed high-throughput transcriptome sequencing of paired ES-LUAD and HC tissues. A total of 76 samples were included in the analysis after quality control (HC: 39; ES-LUAD: 37). The principal component analysis (PCA) revealed a distinct distribution pattern between patients with ES-LUAD and HCs (Additional file 1: Fig. S5C). We identified 1452 DEGs between patients with ES-LUAD and HCs (|log2FC|> 1), comprising 811 upregulated and 641 downregulated DEGs in ES-LUAD (Additional file 1: Fig. S5B, D). Functional enrichment analysis of DEGs in the KEGG database revealed significant dysregulation of cytokine-cytokine receptor interaction, cell adhesion molecules, tyrosine metabolism, ECM-receptor interaction, mucin-type o-glycan biosynthesis, and glutathione metabolism in patients with ES-LUAD. Simultaneously, GO functional analysis indicated that there were changes in categories such as extracellular matrix, cell communication, and collagen-containing extracellular matrix (Fig. 5A). Thus, while biological processes across different functional databases may vary, the regulation of cell adhesion, extracellular matrix, and interactions among cytokines appear to be key pathways of concurrent changes in microbial and host functional genes (Figs. 4A, 5A).

The dynamic interactions were established to illustrate the relationship between all host DEGs, significant microbiota, and KO genes to assess the multi-omics signatures. First, we utilized WGCNA to conduct feature selection on DEGs for screening clinically relevant genes. Nine modules were identified with a soft threshold power of 8, suggesting a guaranteed of a scale-free network (R2 = 0.85) (Additional file 1: Fig. S6A–E). As a result, a strong positive correlation (rho: 0.42–0.9, p < 0.001) was observed within the brown module, concurrently identifying 277 genes (Additional file 1: Fig. S6F). The heatmap depicts the characteristics of the brown module (Additional file 1: Fig. S6G). We next used Spearman rank correlation analysis to assess the correlation between the microbiome and host transcription factors, showcasing representative significant microbiota, microbiota-related KO genes, and DEGs. The network primarily comprises three main nodes (*M. discipulorum*, [histone H4]-lysine20 N-methyltransferase SETD8, histone H2A), with the nodes containing *M. discipulorum* and [histone H4]-lysine20 N-methyltransferase SETD8 respectively associated with 59 and 35 transcription factors, showing significant positive and negative correlations with transcription factors, respectively (Fig. 5B). Previous studies have reported that the glycosyltransferase ST6GALNAC1 can promote the synthesis of various tumor-associated MUC1-sialyl-Tn glycans [41, 42]. Our results show a significant positive correlation between ST6GALNAC1 and MUC1 (rho: 0.66, Fig. 5C). Additionally, we observed significant positive and negative correlations between ST6GALNAC1 and MUC1 with *M. discipulorum* and [histone H4]-lysine20 N-methyltransferase SETD8, respectively (Fig. 5B, C). These results suggest that *M. discipulorum* and microbiota-related histone modifications may be involved in the regulation of mucosal homeostasis mediated by ST6GALNAC1 and MUC1, potentially contributing to tumorigenesis. This evidence supports an association between changes in lung microbiota and host transcriptional profiles, highlighting a crucial direction for future research to elucidate the mechanistic role of these key microbial species in host physiology.

### Validation of multi-omics signatures to identify prognostic biomarkers

**Fig. 6:**
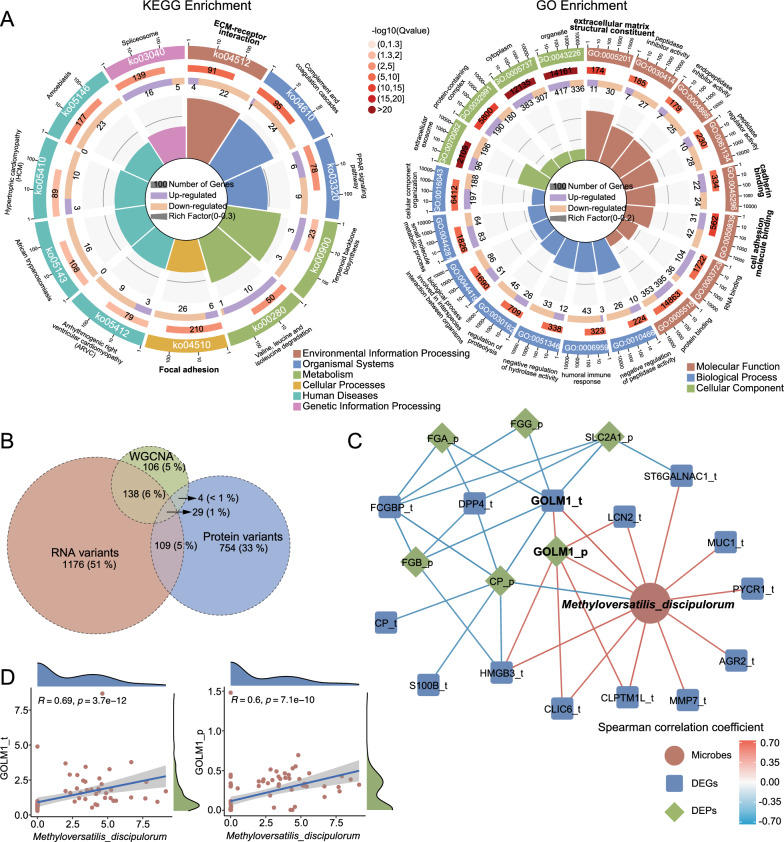
Integrating metagenomics, transcriptomics, and proteomics to identify biomarkers. **A** KEGG and GO pathway enrichment analysis of DEPs between tumor and HC tissues.** B** Venn diagram illustrating the intersection between DEGs and DEPs after filtering by WGCNA signature. **C** Network diagram depicting correlations among signature microbial, DEGs, and DEPs (p < 0.05). Microbes, DEGs, and DEPs are represented by orange circles, blue squares, and green diamonds respectively. Color scale represents the Spearman correlation coefficient. Only correlation pairs containing microbes with |rho|> 0.6 and p < 0.05 were visualized. **D** Scatter plot representing the correlation between *M. discipulorum* and GOLM1 at the transcriptional and protein levels

Gene expression is regulated by transcriptional and translational processes, which may be influenced by post-transcriptional regulation, mRNA stability, and the translation rate [43]. To complement the transcriptomic differences and gain a comprehensive understanding, we conducted proteomic analysis on samples corresponding to the metagenomics sequencing. A partial but significant distinction was observed between the patients with ES-LUAD and HCs (Additional file 1: Fig. S6C). We identified 896 significantly changed DEPs, including 467 that are upregulated and 429 that are downregulated, between the patients with ES-LUAD and HCs (|log2FC|> 1), as illustrated in the volcano plot and heatmap (Additional file 1: Fig. S6B, D). Furthermore, KEGG analysis revealed that the DEPs were enriched in ECM–receptor interaction, focal adhesion, and the PPAR signaling pathway. Significantly enriched GO terms included extracellular matrix structural constituent, cell adhesion molecule binding, small molecule metabolic process, and cadherin binding (Fig. 6A). Regarding the functional enrichment results of the metagenome and transcriptome, we found that the multi-omics datasets revealed key pathways alterations in pathways or components related to ECM–receptor interaction, focal adhesion, and the extracellular matrix (Figs. 4A, 5A, 6A).

**Fig. 7:**
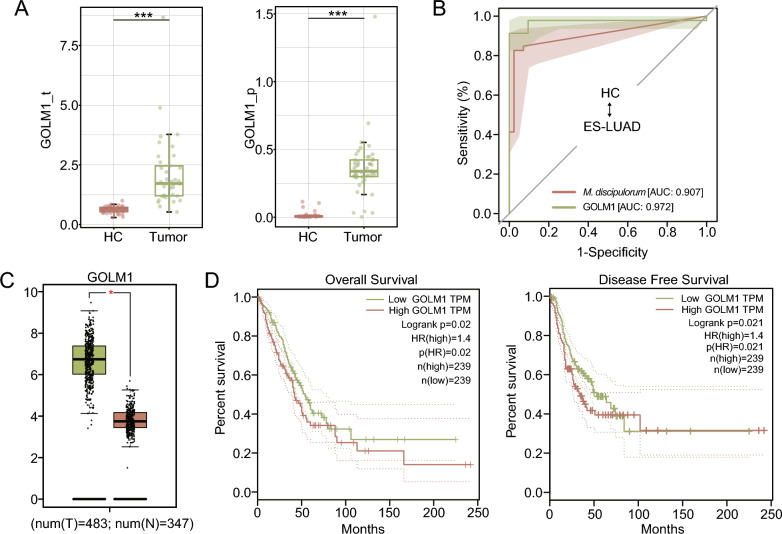
Identifying biomarkers associated with ES-LUAD prognosis. **A** Boxplot showing the relative expression of GOLM1 at the transcriptional and protein levels between patients with ES-LUAD and HCs. **B** ROC curve of *M. discipulorum* and GOLM1. **C** The expression of GOLM1 in GEPIA2. **D** Cox hazards models based on GEPIA 2

Next, we employed proteomics to further validate the findings of the transcriptomics. Twenty-nine DEGs/DEPs exhibited differences concurrently in both the transcriptome and proteome, although some may have displayed opposing expression trends (Fig. 6B, Additional file 1: Fig. S8). We then constructed a network to illustrate the co-occurrence correlation among metagenome, transcriptome, and proteome features. These strong correlations mostly appear as positive associations between metagenome and transcriptome features, with negative associations between the transcriptome and proteome (Fig. 6C). Among the strong correlations in *M. discipulorum*, we observed a prominent trend where the increase in the abundance of *M. discipulorum* was accompanied by the expression of GOLM1 at both the transcript and protein levels (Fig. 6C). Crucially, all three exhibited a significant positive correlation. Golgi membrane protein 1 (GOLM1) has been reported to participate in modulating the immunosuppressive microenvironment and it has been implicated in the onset and progression of hepatocellular carcinoma, glioblastoma, and melanoma [44–46]. The expression of GOLM1 in transcriptomics and proteomics is depicted in Fig. 7A, showing significant differences between patients with ES-LUAD and HCs. As expected, both GOLM1 (AUC: 0.972) and *M. discipulorum* (AUC: 0.907) could be used to effectively differentiate between ES-LUAD and HCs (Fig. 7B). To mitigate the limitations arising from small sample sizes, the results from GEPIA data also validated our findings (Fig. 7C). Furthermore, Cox hazard models based on GEPIA indicated that high GOLM1 expression was associated with shorter OS and DFS (Fig. 7D). Thus, the biological processes involving *M. discipulorum* and GOLM1 may be crucial components of ES-LUAD initiation, while GOLM1 could also guide the prognosis of patients with ES-LUAD.

### Discrimination of patients with ES-LUAD using machine learning

**Fig. 8:**
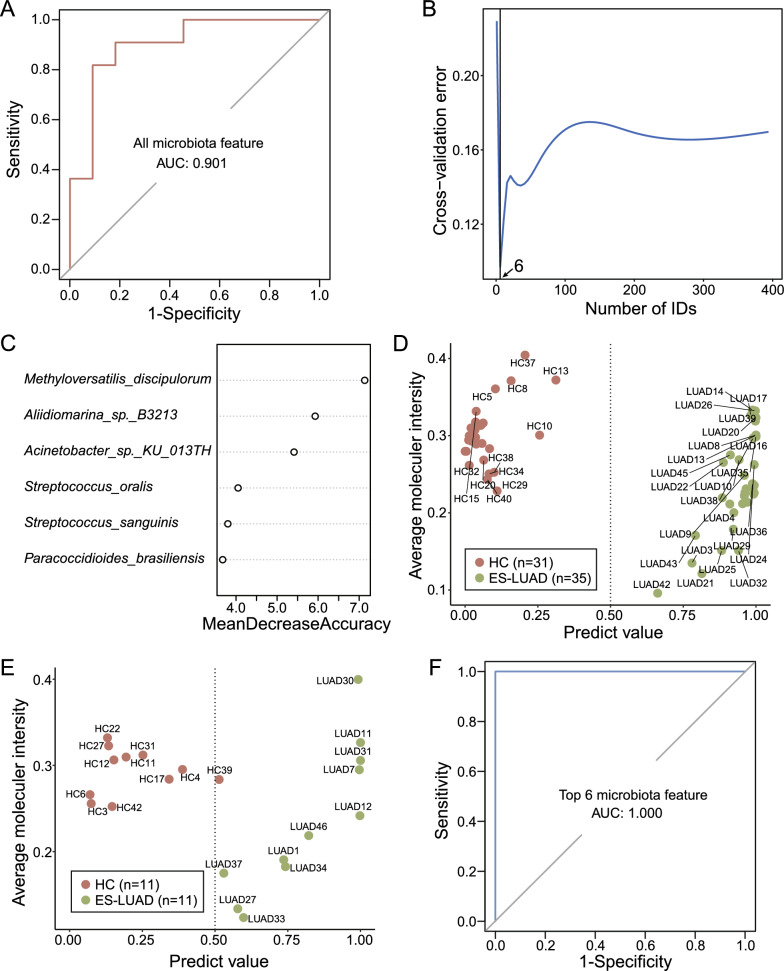
ES-LUAD classifier based on the signatures of tissue microbiome. **A** Validation queue ROC curve for establishing a random forest model based on 398 microbes (training AUC = 1). **B** Selection of optimal feature count based on tenfold cross-validation. **C** Feature ranking based on importance values. **D** Performance of the RF model in the training queue. **E** Performance of the RF model in the validation queue. **F** Validation queue ROC curves of the top six bacteria (training AUC = 1)

To assess the potential value of signature microbiota, microbial KO genes, mRNA, and protein features as diagnostic markers, random forest (RF) classifier models with 10 repeats of tenfold cross-validation were constructed to distinguish patients with ES-LUAD and HCs. A total of 46 patients with ES-LUAD and 42 HCs were divided into training and validation sets at a ratio of 75% and 25%, respectively. Initially, we constructed an RF model using all microbial features, which yielded an area under the curve (AUC) value of 0.901 in the validation set (Fig. 8A). After tenfold cross-validation, the minimum cross-validation error occurred with six features (Fig. 8B). The priority order of these six bacteria was as follows: *M. discipulorum*, *Aliidiomarina sp. B3213*, *Acinetobacter sp. KU 013TH*, *Streptococcus oralis*, *Streptococcus sanguinis*, and *Paracoccidioides brasiliensis* (Fig. 8C). Further testing of this RF model composed of these six bacteria demonstrated excellent diagnostic performance in the training and validation sets (Fig. 8D, E). This model exhibited superior discrimination ability between patiens with ES-LUAD and HCs compared to models based on all microbial features (AUC: 1.000) (Fig. 8F).

The models based on protein and mRNA features were also well performed, with AUC values of 0.934 and 0.967, respectively. However, the feature selection results indicated lower cross-validation errors and better discrimination abilities with 150 proteins or 500 mRNAs, which contradicted the principle of constructing simple models (Additional file 1: Fig. S10A, B). The RF model based on KOs showed an AUC of 0.694, with a high cross-validation error (Additional file 1: Fig. S10C).

Next, we aimed to establish a multi-omics classifier to achieve optimal ES-LUAD diagnostic results. Since mRNAs and KOs performed poorly in model classification, we chose microbial and protein features to construct a multi-omics classifier, which demonstrated excellent diagnostic efficacy (AUC: 0.942). However, the lowest cross-validation error occurred with 45 features, and after tenfold cross-validation, the AUC in the validation set was 0.909 (Additional file 1: Fig. S10D). In comparison, the RF model based on six bacterial features proved to be more accurate in distinguishing between HCs and patients with ES-LUAD than the combined model. Therefore, as a promising non-invasive tool, the bacterial marker panel holds great potential for detecting and distinguishing patients with ES-LUAD and healthy individuals.

## Discussion

Our prospective study results revealed a significant association between local microbiota in lower respiratory tissues and dynamic changes in host transcription and translation, which may be relevant to the pathogenesis of ES-LUAD. To the best of our knowledge, this is the first comprehensive study employing ultra-deep metagenomics, host transcriptomics, and proteomics to identify the ES-LUAD lung tissues microbiota, including bacteria, fungi, and viruses. The limited biomass in lung tissue together with our limited knowledge has led us to overlook the role of microbiota in maintaining the mucosal immune balance in the lower respiratory tract. In our study, we systematically eliminated all potential confounding factors that might affect the lung microbiota, including environmental habitat, smoking, alcohol consumption, antibiotic use, inhaled steroid nebulization, and lung infection. Moreover, our samples were isolated from any contamination from oral and upper respiratory microbiota and swiftly collected under sterile and dust-free conditions, ensuring an exceptionally high level of confidence in our data. We observed that the microbial load in tumor tissues was similar to that in NAT but significantly lower than that in HC, providing similar evidence from previous research [40]. Owing to anatomical proximity, bacteria within the tumor may also originate from the NAT, providing an explanation for the observed high similarity between the microbial compositions of the tumor and paired NAT samples. Previous studies using 16S rRNA profiling identified predominant lung tissue microbiota within the phyla *Proteobacteria*, *Actinobacteria*, *Firmicutes*, and *Bacteroidetes* [18]. Similarly, our research confirmed these findings, although we observed a higher abundance of *Ascomycota* than *Bacteroidetes*, a consistent pattern observed across almost all samples. Notably, compared to HCs, patients with ES-LUAD exhibit a reduced species abundance but a high prevalence of rare species with low individual counts yet high diversity. Moreover, the decreased ratio between Firmicutes and Bacteroidetes signifies an imbalance in the local microbiota of ES-LUAD [39, 47].

The microenvironment of ES-LUAD involves shared alterations in multiple distinct taxonomic groups and microbial functional genes. *M. discipulorum* is predominantly isolated from natural environments [48]; however, our data indicate its significant involvement in the modulation of diverse microenvironments, although its pathogenic potential remains unknown. *E. faecium* has garnered significant attention in tumor immunotherapy recently due to its ability to enhance T-cell responses and improve the efficacy of anti-PD-L1 antitumor treatments [49, 50]. Although *H. pylori* is widely recognized as a risk factor for gastric cancer, emerging evidence suggests an association between *H. pylori* infection and an increased risk of lung cancer [51]. Intriguingly, our dataset revealed a lower abundance of *E. faecium* and *H. pylori* in the group with cancer than HC. Indeed, as these species may demonstrate varying degrees of pathogenicity in different diseases, the correlation with lung cancer remains to be explored further.

Significant correlations were also observed between characteristic changes in microbial abundance, particularly in ES-LUAD and NAT samples, and the regulation of ribosomal and histone genes. Aberrant ribosomes may undergo degradation by quality control mechanisms, leading to an overall reduction in protein synthesis rates. Should aberrant ribosomes escape quality control mechanisms, they may facilitate decreased translation fidelity or altered mRNA translation patterns, potentially contributing to tumorigenesis [52, 53]. Furthermore, these abnormalities may induce host inflammatory responses, influencing gene expression by modifying the cellular environment, impacting signaling pathways, and activating immune cells, or by suppressing the host’s immune response to facilitate tumor escape and progression [54]. Epigenetic modifications, particularly alterations in histone proteins, play a crucial role in gene expression regulation and cancer development [55, 56]. The study found upregulation of histone H2A and histone H3, accompanied by downregulation of histone H4 lysine20 N-methyltransferase SETD8 in ES-LUAD. This suggests a shift in the epigenetic landscape favoring tumor progression. Correlation analysis also indicated potential interactions between microbial communities and histone modifications, highlighting the intricate interplay between the microbiota and epigenetic regulation in cancer. The findings suggest that functional alterations in the lung tissue metagenome, driven by dysbiosis and microbial dysregulation, may play a specific role in the etiology of ES-LUAD [57].

In this study, we have uncovered intriguing connections between *M. discipulorum*, ST6GALNAC1, MUC1, and host physiology, particularly in the context of tumorigenesis. Our findings reveal a significant positive correlation between ST6GALNAC1, an enzyme implicated in the synthesis of tumor-associated glycans, and MUC1, a mucin glycoprotein frequently dysregulated in cancer. Chronic inflammation promotes abnormal MUC1 glycosylation in epithelial cells, while the inflammatory microenvironment can also induce changes in cell polysaccharide composition by regulating glycosyltransferases [58, 59]. During chronic inflammation and carcinogenesis, M2 macrophages induce the expression of ST6GALNAC1, thereby promoting abnormal glycosylation of MUC1, leading to the formation of tumor-associated sialyl-Tn (sTn) O-glycans, exacerbating disease progression [42]. Additionally, ST6GALNAC1 and MUC1 collectively participate in the expression of tumor-associated glycan antigen STn in cancer cells, potentially affecting dendritic cell maturation and thus influencing immune responses [41]. Furthermore, our analysis highlights the involvement of *M. discipulorum* and microbiota-related histone modifications in this regulatory network. Specifically, we observe significant correlations between *M. discipulorum*, ST6GALNAC1, MUC1, and specific microbiota-related function genes, underscoring the intricate interplay between microbial species and host gene expression profiles. Notably, the presence of *M. discipulorum* and specific microbiota-related histone modifications appears to modulate the relationship between ST6GALNAC1, MUC1, and host transcriptional activity. Thus, exploring how alterations in the lung microbiota influence the expression and activity of key oncogenic pathways, such as those involving ST6GALNAC1 and MUC1, may unveil novel targets for therapeutic intervention.

The integration of multi-omics data, including metagenomics, transcriptomics, and proteomics, provides a comprehensive understanding of the molecular landscape underlying disease processes. Our findings revealed a notable convergence of differential expression patterns between *M. discipulorum* and GOLM1 across both transcriptomic and proteomic levels. Previous research has shown that abnormal regulation of GOLM1 plays a significant role in the development and metastasis of colorectal cancer, potentially promoting tumor immune evasion and metastasis by recruiting myeloid-derived suppressor cells [60]. However, GOLM1 also maintains the homeostasis of intestinal epithelial cells by regulating the Notch signaling pathway, protecting the intestine from damage caused by colitis and colorectal cancer [61]. In Crohn's disease, there is abnormal interaction between microbiota and host proteins such as GOLM1, which may be related to the development of intestinal inflammation and bacterial translocation [62]. In hepatocellular carcinoma, GOLM1 promotes the stability of PD-L1 and transports PD-L1 into tumor-associated macrophage-derived exosomes, leading to suppression of CD8 + T cells, revealing its role in regulating the immunosuppressive environment [44]. Additionally, GOLM1 can also be used for early diagnosis of hepatocellular carcinoma [63, 64]. Therefore, GOLM1 has a diverse role in various malignancies, including maintenance of inflammation, tumor initiation and metastasis, as well as early diagnosis. Our study extends this understanding by elucidating its potential relevance in ES-LUAD. Furthermore, the association between high GOLM1 expression and unfavorable clinical outcomes, as indicated by shorter OS and DFS, underscores its prognostic significance in ES-LUAD. This highlights GOLM1 as a potential biomarker for risk stratification and therapeutic decision-making in patients with ES-LUAD. Moreover, the identification of *M. discipulorum* as a co-occurring factor further emphasizes the intricate crosstalk between the microbiome and host biology in cancer development. The observed associations suggest a potential mechanistic link between microbial dysbiosis and GOLM1-mediated immune modulation, contributing to the pathogenesis of ES-LUAD.

The results of our study underscore the potential of utilizing microbial, microbial-associated KO genes, mRNA, and protein features as diagnostic markers for discriminating between patients with ES-LUAD and HCs. Using cross-validated random forest classifier models, we successfully distinguished patients with ES-LUAD and HCs, particularly highlighting the efficacy of a model incorporating six specific bacterial features. Interestingly, the RF model based on microbial features outperformed those based on protein and mRNA features, highlighting the potential superiority of microbial signatures in ES-LUAD diagnosis. We further aimed to establish a multi-omics classifier combining microbial and protein features to optimize diagnostic accuracy. While this multi-omics approach showed promising diagnostic efficacy, it became evident that the RF model based on six bacterial features outperformed the multi-omics classifier composed of 45 features in distinguishing between patients with ES-LUAD and HCs. However, further validation of our findings in larger cohorts and across diverse populations is warranted to confirm the robustness and generalizability of the microbial marker panel.

The highlights of this study lie in the use of ultra-deep metagenomics in conjunction with transcriptomics and proteomics to explore the microbial changes in ES-LUAD and assess the feasibility of these changes as a non-invasive diagnostic method. Furthermore, this research represents one of the few investigations into the microbiome of ES-LUAD, specifically among non-smokers. Despite the careful design and rigorous sample inclusion, our study still has several limitations. First, as a cross-sectional study, our sample size is relatively limited, and there is a lack of external cohort validation. Validation of our conclusions requires a multicenter approach with a large sample size. Second, despite efforts to mitigate differences related to sex and age, confounding factors such as individual variations remain unavoidable. Third, our detection techniques are primarily designed for microbial communities and may not be universally applicable when identifying lower abundance classifications such as fungi, viruses, or archaea. Future work will involve mechanistic experiments to elucidate specific causal relationships.

## Conclusions

In summary, our study elucidates distinctive changes in the local microbiome of ES-LUAD and its dynamic interplay with the host, contributing to an enhanced understanding of its pathogenic mechanisms. The potential biomarkers identified through multi-omics approaches offer promising avenues for the development of non-invasive tools to assist in the screening of ES-LUAD. In the long term, our study lays the foundation for larger-scale validation experiments, contributing to the identification of novel microbial diagnostic targets for ES-LUAD.

## Supplementary Information

**Additional file 1: Figure S1.** Quality control of metagenomic data. **A** Comparison of quality control passed reads after filtering in 129 samples. **B** Specaccum species accumulation curves of three groups. **C** Rarefaction curves showing observed species richness taken from the 129 samples. **D** Overall taxa distribution of the microbiome kingdom in three groups. **Figure S2.** Microbial compositions in the cohort. Microbial compositions of the patients with ESLUAD and HCs at the phylum (**A**), genus (**B**), and species (**C**) levels. The top 10/20 abundant microbial taxa are shown with different gradient colors. The microbial composition is arranged in order of the mostabundant taxonomic ranks. **Figure S3.** Representative microbes exhibiting significant alterations between patients with ESLUAD and HCs. *** p < 0.001 as determined by Kruskal–Wallis test. **Figure S4.** Correlation between intrapulmonary microbiota and clinical features. **A**, **D** Comparison of the alpha diversity (Chao1/Shannon/Simpson index) and beta diversity (Bray–Curtis distance) at the species level with tumor infiltration in patients with ES-LUAD. **B**, **E** Comparison of the alpha diversity (Chao1/Shannon/Simpson index) and beta diversity (Bray–Curtis distance) at the species level with solid component of tumor in patients with ES-LUAD. C, F Comparison of the alpha diversity (Chao1/Shannon/Simpson index) and beta diversity (Bray–Curtis distance) at the species level with multiple-primary nodules in patients with ES-LUAD. Box plots show median ± quartiles, and the whiskers extend from the hinge to the largest or smallest value no further than 1.5-fold of the interquartile range. ns: Not significant, p-value as determined by Wilcoxon rank-sum test. AIS: Adenocarcinoma in situ, MIA: Minimally invasive adenocarcinoma, IA: Invasive adenocarcinoma, pGGN: Pure ground glass nodules, mGGN: Mixed ground glass nodules, SN: Solid nodule. **Figure S5.** Overview of transcriptome data. **A** RNA-Seq passed reads sequenced by Illumina NoveSeq 6000 Nanopore platforms (Wilcoxon rank-sum test). **B** Clustering heatmap of the DEGs between patients with ES-LUAD and HCs (DESeq2, |log2FC| > 1). **C** PCA analysis reveals differences in the transcriptomes of patients with ES-LUAD and HCs. **D** Volcano diagram shows the significant DEGs between patients with ES-LUAD and HCs (DESeq2, |log2FC| > 1). **Figure S6.** Identification of ES-LUAD-related mRNAs in the transcriptome dataset through WGCNA. **A**–**D** Network fitting calculations with fitted curves for selected network construction parameters. **A** Correlation coefficient corresponding to different power. **B** Average connectivity of the network constructed with different power values. When the power is taken as 8, the correlation coefficient is higher, and the average connectivity of the network is also higher, so the value of power used in the construction of the subsequent module is 8. **C** The distribution of network connectivity when the power is 8; **D** The test result of the power law distribution. As can be seen from the figure, k and p(k) are negatively correlated (correlation coefficient: 0.85), indicating that the selected power value enables the establishment of a scale-free network of genes. **E** The result of weighted co-expression network construction. **F** Heatmap of correlation analysis between modules and clinical traits. **G** Gene expression information statistics within modules. **Figure S7.** Overview of proteomic data. **A** QC sample correlation represents the process stability. **B** Clustering heatmap of the DEPs between patients with ES-LUAD and HCs (Wilcoxon rank-sum test, log2 fold change > 1). **C** PCA reveals differences in the proteome of patients with ES-LUAD and HCs. **D** Volcano diagram shows the significant DEPs between patients with ES-LUAD and HCs (Wilcoxon rank-sum test, log2 fold change > 1). **Figure S8.** Validation and prognostic information of DEGs and DEPs in public databases. The top represents the expression of DEGs, and the bottom represents the expression of DEPs. The middle represents the OS and DFS. Solid lines indicate significance at p < 0.05 (Mantel–Cox test). **Figure S9.** Random forest model based on multi-omics data. **A** The left panel represents the validation queue ROC curve for the random forest model established based on 3000 proteins (training AUC = 1). The middle panel depicts the selection of optimal feature count based on 10-fold cross-validation. The right panel shows the ROC curve for the top 150 proteins in the validation queue (training AUC = 1). **B** The left panel represents the validation queue ROC curve for the random forest model established based on 13846 mRNAs (training AUC = 1). The middle panel depicts the selection of optimal feature count based on 10-fold cross-validation. The right panel shows the ROC curve for the top 500 mRNAs in the validation queue (training AUC = 1). **C** The left panel represents the validation queue ROC curve for the random forest model established based on 196 KO genes (training AUC = 1). The middle panel depicts the selection of optimal feature count based on 10-fold cross-validation. **D** The left panel represents the validation queue ROC curve for the random forest model established based on 398 microbes and 3000 proteins (training AUC = 1). The middle panel depicts the selection of optimal feature count based on 10-fold cross-validation. The right panel shows the ROC curve for the top 45 microbes and proteins in the validation queue (training AUC = 1).

## Data Availability

The raw sequence data reported in this paper have been deposited in the Genome Sequence Archive (Genomics, Proteomics & Bioinformatics 2021) in National Genomics Data Center (Nucleic Acids Res 2022), China National Center for Bioinformation/Beijing Institute of Genomics, Chinese Academy of Sciences (GSA-Human: HRA006270 and HRA006254, and OMIX: OMIX005363, for metagenomics, transcriptomics, and proteomics, respectively.) that are publicly accessible at https://ngdc.cncb.ac.cn/gsa-human and https://ngdc.cncb.ac.cn/omix [65].

## Abbreviations

ES-LUAD: Early-stage lung adenocarcinoma
HC: Healthy control
NSCLC: Non-small-cell lung cancer
SCC: Squamous cell carcinoma
BALF: Bronchoalveolar lavage fluid
PSP: Primary spontaneous pneumothorax
LC–MS/MS: Liquid chromatography–tandem mass spectrometry
NAT: Normal adjacent tissue
IASLC: International Association for the Study of Lung Cancer
LDA: Linear discriminant analysis
LEfSe: Linear discriminant analysis effect size
DEG: Differentially expressed gene
WGCNA: Weighted Gene Co-expression Network Analysis
GdmCl: Guanidinium hydrochloride
TCEP: Tris(2-carboxyethyl)phosphine
ACN: Acetonitrile
TFA: Trifluoroacetic acid
FA: Formic acid
DDA: Data-dependent acquisition
HCD: Higher energy collision dissociation
FDR: False discovery rate
DEP: Differentially expressed protein
ROC: Receiver operating characteristic curves
GEPIA2: Gene expression profiling interactive analysis 2
TCGA: The cancer genome atlas
GTEx: Genotype-tissue expression
OS: Overall survival
DFS: Disease-free survival
PCoA: Principal coordinate analysis
KO: Kegg orthology
GO: Gene ontology
RP: Ribosomal protein
PCA: Principal component analysis
GOLM1: Golgi membrane protein 1
AUC: Under the curve
