# Correction: Integrative metagenomic, transcriptomic, and proteomic analysis reveal the microbiota-host interplay in early-stage lung adenocarcinoma among non-smokers

**DOI:** 10.1186/s12967-025-06713-x

**Published:** 2025-08-15

**Authors:** Yaohui Sun, Zhiming Gan, Xiaojin Wang, Jian Liu, Wei Zhong, Zhiyan Zhang, Jiebin Zuo, Hang Zhong, Xiuting Huang, Zhixiang Yan, Qingdong Cao

**Affiliations:** 1 Department of Thoracic Surgery and Lung Transplantation, The Fifth Affiliated Hospital of Sun Yat-Sen University, Zhuhai, 519000 Guangdong China; 2 Guangdong Provincial Key Laboratory of Biomedical Imaging and Guangdong Provincial Engineering Research Center of Molecular Imaging, The Fifth Affiliated Hospital of Sun Yat-Sen University, Zhuhai, 519000 Guangdong China; 3 Cardiovascular Disease Center, The Fifth Affiliated Hospital of Sun Yat-Sen University, Zhuhai, 519000 Guangdong China

**Correction:** ***J Transl Med*** **22, 652 (2024)**

10.1186/s12967-024-05485-0

Following publication of the original article [1], the authors reported an error in the Dataset ID.

The dataset IDs for the raw sequence data have now been corrected to:

HRA006254 (Submission ID: HRA008925)

HRA006270 (Submission ID: HRA008921)

The original article [1] has been updated.
