# Supplementary figures and images for: Integrative metagenomic, transcriptomic, and proteomic analysis reveal the microbiota-host interplay in early-stage lung adenocarcinoma among non-smokers
Source: J Transl Med. 2024 Jul 13;22:652. doi: 10.1186/s12967-024-05485-0 (PMC11245786; doi:10.1186/s12967-024-05485-0)

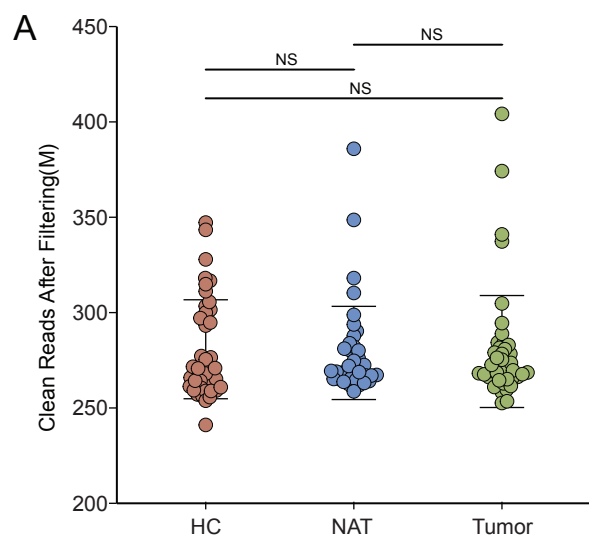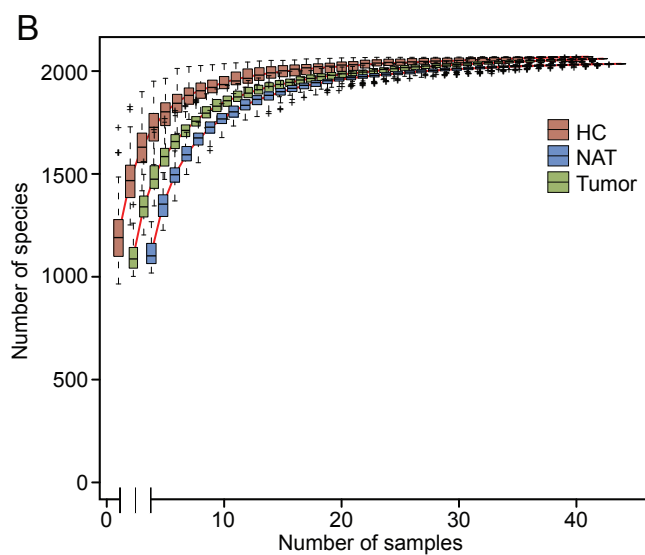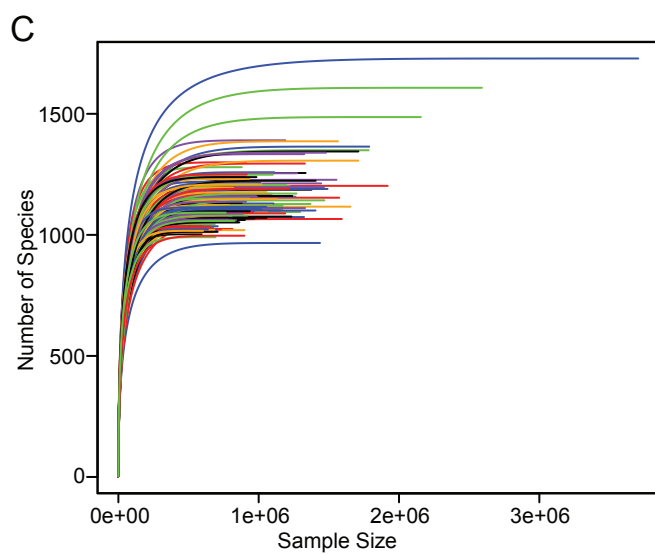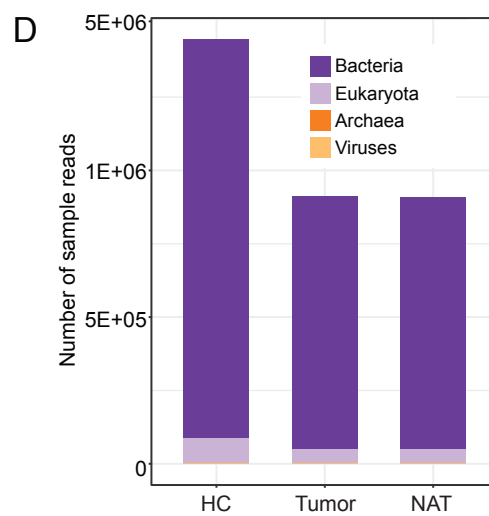

Supplement: Supplementary file 1 — Additional file 1: Figure S1. Quality control of metagenomic data. A Comparison of quality control passed reads after filtering in 129 samples. B Specaccum species accumulation curves of three groups. C Rarefaction curves showing observed species richness taken from the 129 samples. D Overall taxa distribution of the microbiome kingdom in three groups. Figure S2. Microbial compositions in the cohort. Microbial compositions of the patients with ESLUAD and HCs at the phylum (A), genus (B), and species (C) levels. The top 10/20 abundant microbial taxa are shown with different gradient colors. The microbial composition is arranged in order of the mostabundant taxonomic ranks. Figure S3. Representative microbes exhibiting significant alterations between patients with ESLUAD and HCs. *** p < 0.001 as determined by Kruskal–Wallis test. Figure S4. Correlation between intrapulmonary microbiota and clinical features. A, D Comparison of the alpha diversity (Chao1/Shannon/Simpson index) and beta diversity (Bray–Curtis distance) at the species level with tumor infiltration in patients with ES-LUAD. B, E Comparison of the alpha diversity (Chao1/Shannon/Simpson index) and beta diversity (Bray–Curtis distance) at the species level with solid component of tumor in patients with ES-LUAD. C, F Comparison of the alpha diversity (Chao1/Shannon/Simpson index) and beta diversity (Bray–Curtis distance) at the species level with multiple-primary nodules in patients with ES-LUAD. Box plots show median ± quartiles, and the whiskers extend from the hinge to the largest or smallest value no further than 1.5-fold of the interquartile range. ns: Not significant, p-value as determined by Wilcoxon rank-sum test. AIS: Adenocarcinoma in situ, MIA: Minimally invasive adenocarcinoma, IA: Invasive adenocarcinoma, pGGN: Pure ground glass nodules, mGGN: Mixed ground glass nodules, SN: Solid nodule. Figure S5. Overview of transcriptome data. A RNA-Seq passed reads sequenced by Illumina NoveSeq 6000 Nanopor [file 12967_2024_5485_MOESM1_ESM.zip › Suppl-figs/Figure S1.pdf]

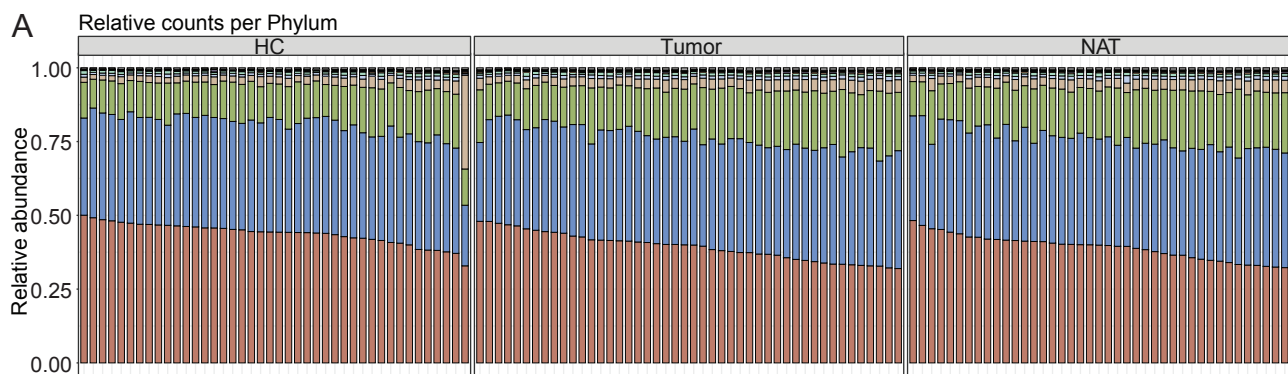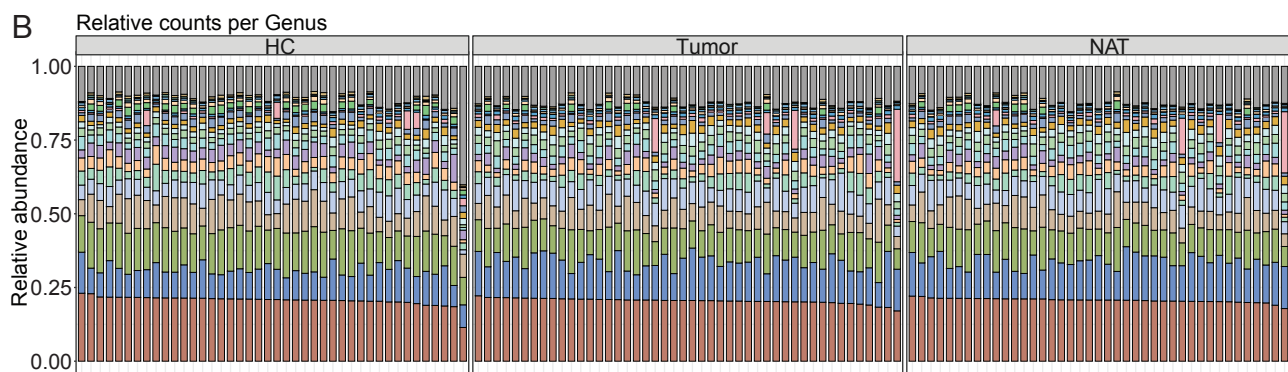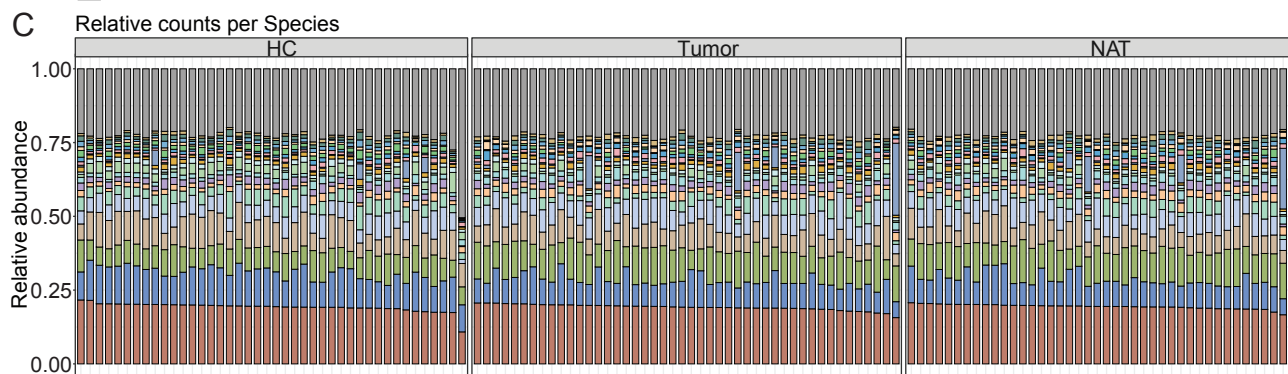

Supplement: Supplementary file 1 — Additional file 1: Figure S1. Quality control of metagenomic data. A Comparison of quality control passed reads after filtering in 129 samples. B Specaccum species accumulation curves of three groups. C Rarefaction curves showing observed species richness taken from the 129 samples. D Overall taxa distribution of the microbiome kingdom in three groups. Figure S2. Microbial compositions in the cohort. Microbial compositions of the patients with ESLUAD and HCs at the phylum (A), genus (B), and species (C) levels. The top 10/20 abundant microbial taxa are shown with different gradient colors. The microbial composition is arranged in order of the mostabundant taxonomic ranks. Figure S3. Representative microbes exhibiting significant alterations between patients with ESLUAD and HCs. *** p < 0.001 as determined by Kruskal–Wallis test. Figure S4. Correlation between intrapulmonary microbiota and clinical features. A, D Comparison of the alpha diversity (Chao1/Shannon/Simpson index) and beta diversity (Bray–Curtis distance) at the species level with tumor infiltration in patients with ES-LUAD. B, E Comparison of the alpha diversity (Chao1/Shannon/Simpson index) and beta diversity (Bray–Curtis distance) at the species level with solid component of tumor in patients with ES-LUAD. C, F Comparison of the alpha diversity (Chao1/Shannon/Simpson index) and beta diversity (Bray–Curtis distance) at the species level with multiple-primary nodules in patients with ES-LUAD. Box plots show median ± quartiles, and the whiskers extend from the hinge to the largest or smallest value no further than 1.5-fold of the interquartile range. ns: Not significant, p-value as determined by Wilcoxon rank-sum test. AIS: Adenocarcinoma in situ, MIA: Minimally invasive adenocarcinoma, IA: Invasive adenocarcinoma, pGGN: Pure ground glass nodules, mGGN: Mixed ground glass nodules, SN: Solid nodule. Figure S5. Overview of transcriptome data. A RNA-Seq passed reads sequenced by Illumina NoveSeq 6000 Nanopor [file 12967_2024_5485_MOESM1_ESM.zip › Suppl-figs/Figure S2.pdf]

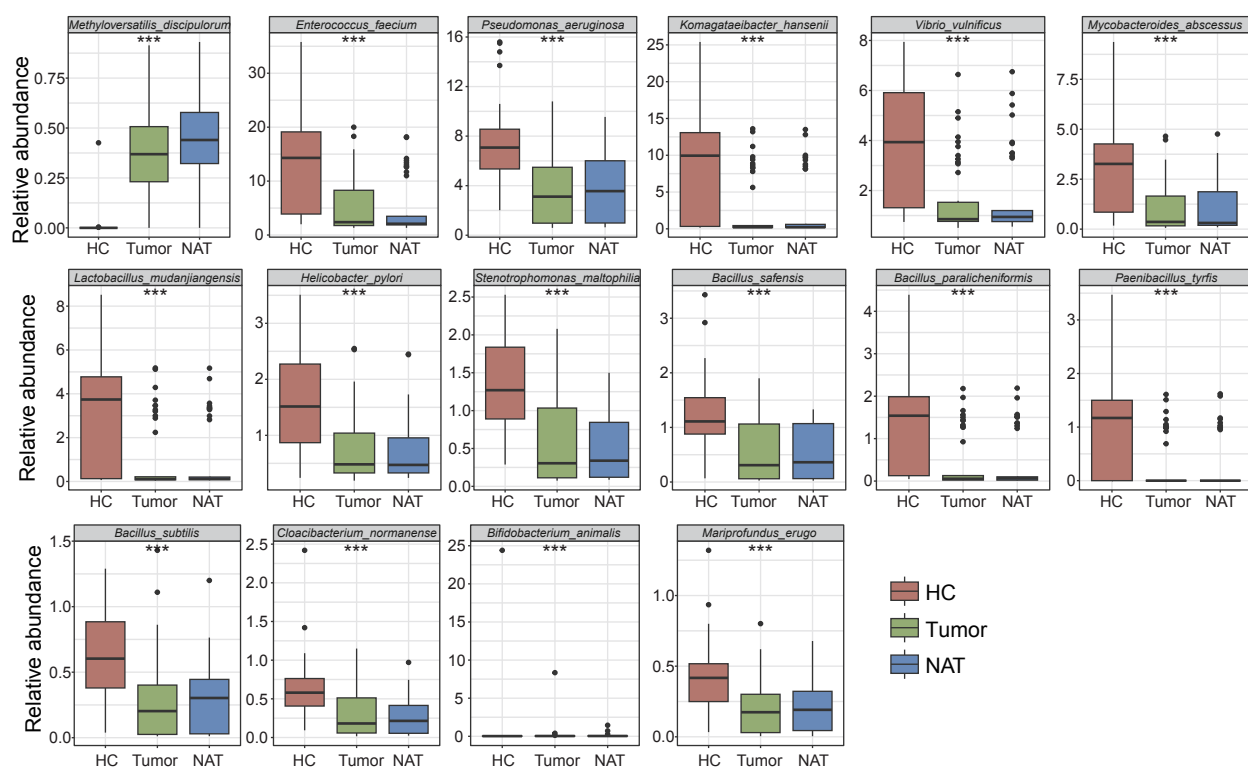

Supplement: Supplementary file 1 — Additional file 1: Figure S1. Quality control of metagenomic data. A Comparison of quality control passed reads after filtering in 129 samples. B Specaccum species accumulation curves of three groups. C Rarefaction curves showing observed species richness taken from the 129 samples. D Overall taxa distribution of the microbiome kingdom in three groups. Figure S2. Microbial compositions in the cohort. Microbial compositions of the patients with ESLUAD and HCs at the phylum (A), genus (B), and species (C) levels. The top 10/20 abundant microbial taxa are shown with different gradient colors. The microbial composition is arranged in order of the mostabundant taxonomic ranks. Figure S3. Representative microbes exhibiting significant alterations between patients with ESLUAD and HCs. *** p < 0.001 as determined by Kruskal–Wallis test. Figure S4. Correlation between intrapulmonary microbiota and clinical features. A, D Comparison of the alpha diversity (Chao1/Shannon/Simpson index) and beta diversity (Bray–Curtis distance) at the species level with tumor infiltration in patients with ES-LUAD. B, E Comparison of the alpha diversity (Chao1/Shannon/Simpson index) and beta diversity (Bray–Curtis distance) at the species level with solid component of tumor in patients with ES-LUAD. C, F Comparison of the alpha diversity (Chao1/Shannon/Simpson index) and beta diversity (Bray–Curtis distance) at the species level with multiple-primary nodules in patients with ES-LUAD. Box plots show median ± quartiles, and the whiskers extend from the hinge to the largest or smallest value no further than 1.5-fold of the interquartile range. ns: Not significant, p-value as determined by Wilcoxon rank-sum test. AIS: Adenocarcinoma in situ, MIA: Minimally invasive adenocarcinoma, IA: Invasive adenocarcinoma, pGGN: Pure ground glass nodules, mGGN: Mixed ground glass nodules, SN: Solid nodule. Figure S5. Overview of transcriptome data. A RNA-Seq passed reads sequenced by Illumina NoveSeq 6000 Nanopor [file 12967_2024_5485_MOESM1_ESM.zip › Suppl-figs/Figure S3.pdf]

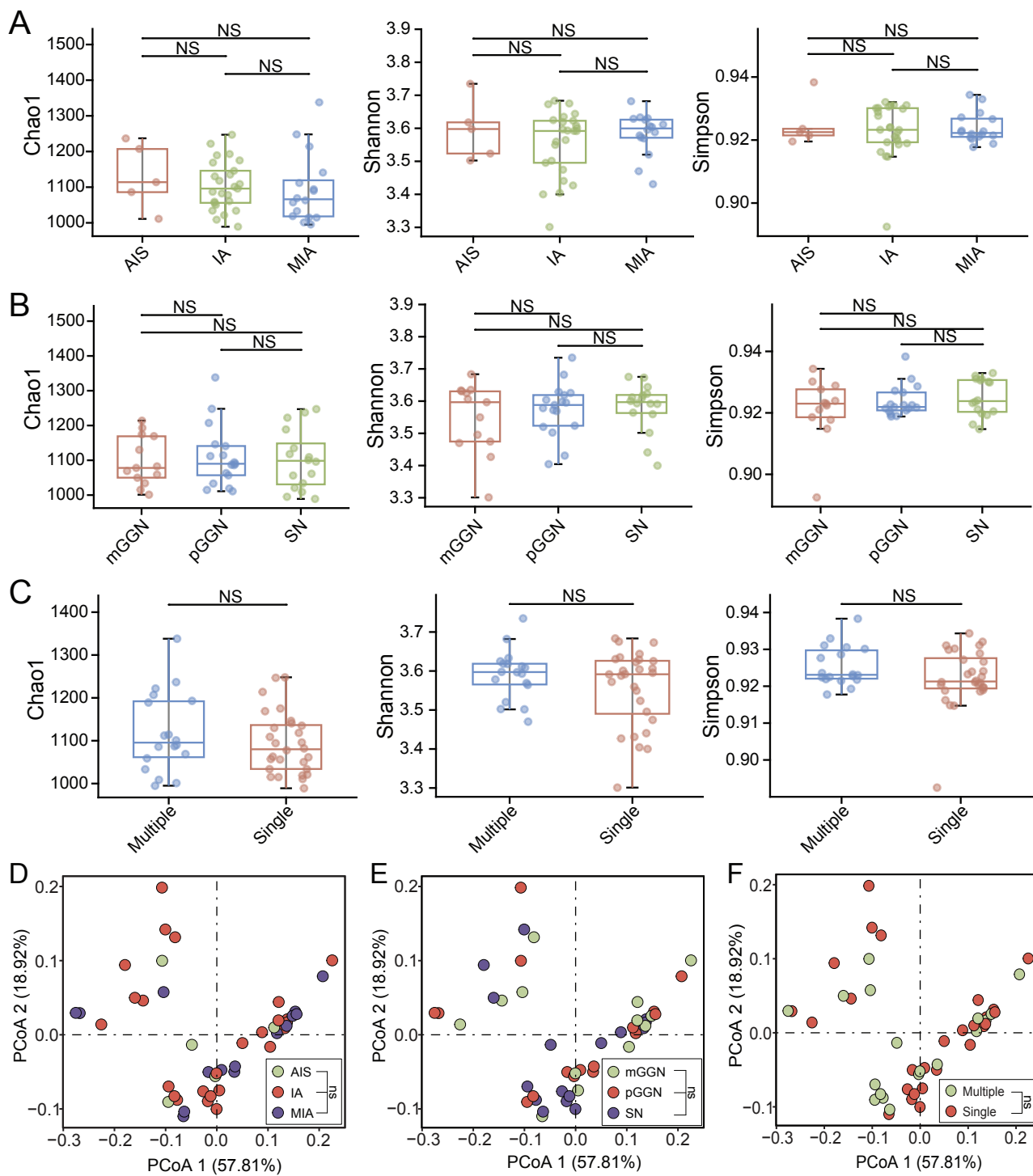

Supplement: Supplementary file 1 — Additional file 1: Figure S1. Quality control of metagenomic data. A Comparison of quality control passed reads after filtering in 129 samples. B Specaccum species accumulation curves of three groups. C Rarefaction curves showing observed species richness taken from the 129 samples. D Overall taxa distribution of the microbiome kingdom in three groups. Figure S2. Microbial compositions in the cohort. Microbial compositions of the patients with ESLUAD and HCs at the phylum (A), genus (B), and species (C) levels. The top 10/20 abundant microbial taxa are shown with different gradient colors. The microbial composition is arranged in order of the mostabundant taxonomic ranks. Figure S3. Representative microbes exhibiting significant alterations between patients with ESLUAD and HCs. *** p < 0.001 as determined by Kruskal–Wallis test. Figure S4. Correlation between intrapulmonary microbiota and clinical features. A, D Comparison of the alpha diversity (Chao1/Shannon/Simpson index) and beta diversity (Bray–Curtis distance) at the species level with tumor infiltration in patients with ES-LUAD. B, E Comparison of the alpha diversity (Chao1/Shannon/Simpson index) and beta diversity (Bray–Curtis distance) at the species level with solid component of tumor in patients with ES-LUAD. C, F Comparison of the alpha diversity (Chao1/Shannon/Simpson index) and beta diversity (Bray–Curtis distance) at the species level with multiple-primary nodules in patients with ES-LUAD. Box plots show median ± quartiles, and the whiskers extend from the hinge to the largest or smallest value no further than 1.5-fold of the interquartile range. ns: Not significant, p-value as determined by Wilcoxon rank-sum test. AIS: Adenocarcinoma in situ, MIA: Minimally invasive adenocarcinoma, IA: Invasive adenocarcinoma, pGGN: Pure ground glass nodules, mGGN: Mixed ground glass nodules, SN: Solid nodule. Figure S5. Overview of transcriptome data. A RNA-Seq passed reads sequenced by Illumina NoveSeq 6000 Nanopor [file 12967_2024_5485_MOESM1_ESM.zip › Suppl-figs/Figure S4.pdf]

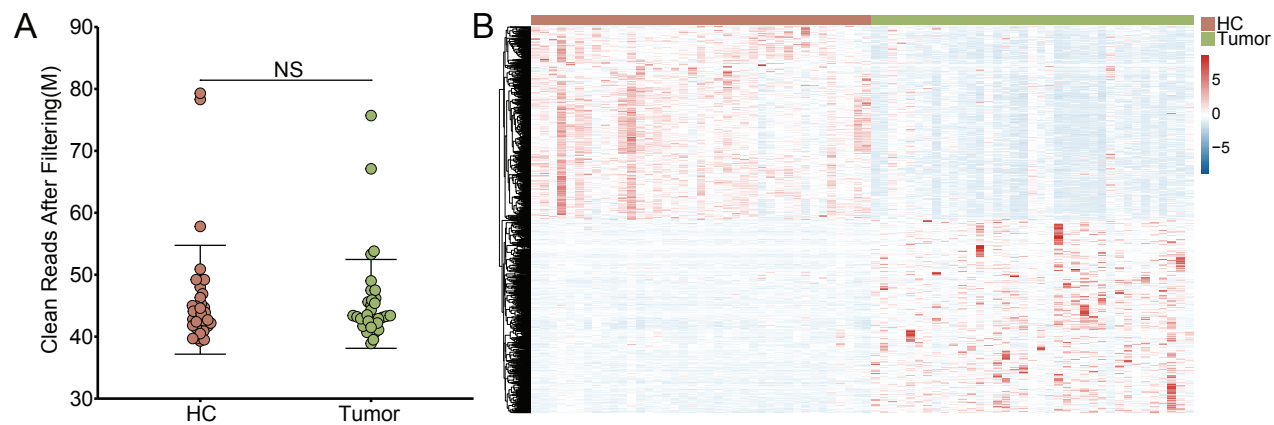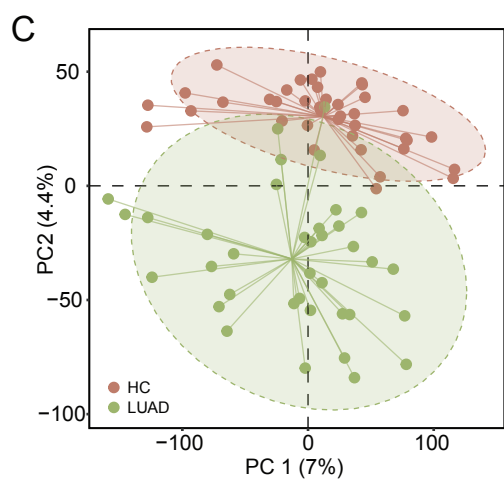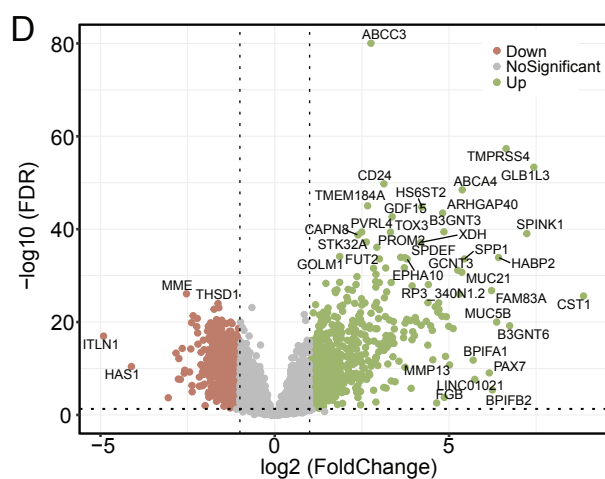

Supplement: Supplementary file 1 — Additional file 1: Figure S1. Quality control of metagenomic data. A Comparison of quality control passed reads after filtering in 129 samples. B Specaccum species accumulation curves of three groups. C Rarefaction curves showing observed species richness taken from the 129 samples. D Overall taxa distribution of the microbiome kingdom in three groups. Figure S2. Microbial compositions in the cohort. Microbial compositions of the patients with ESLUAD and HCs at the phylum (A), genus (B), and species (C) levels. The top 10/20 abundant microbial taxa are shown with different gradient colors. The microbial composition is arranged in order of the mostabundant taxonomic ranks. Figure S3. Representative microbes exhibiting significant alterations between patients with ESLUAD and HCs. *** p < 0.001 as determined by Kruskal–Wallis test. Figure S4. Correlation between intrapulmonary microbiota and clinical features. A, D Comparison of the alpha diversity (Chao1/Shannon/Simpson index) and beta diversity (Bray–Curtis distance) at the species level with tumor infiltration in patients with ES-LUAD. B, E Comparison of the alpha diversity (Chao1/Shannon/Simpson index) and beta diversity (Bray–Curtis distance) at the species level with solid component of tumor in patients with ES-LUAD. C, F Comparison of the alpha diversity (Chao1/Shannon/Simpson index) and beta diversity (Bray–Curtis distance) at the species level with multiple-primary nodules in patients with ES-LUAD. Box plots show median ± quartiles, and the whiskers extend from the hinge to the largest or smallest value no further than 1.5-fold of the interquartile range. ns: Not significant, p-value as determined by Wilcoxon rank-sum test. AIS: Adenocarcinoma in situ, MIA: Minimally invasive adenocarcinoma, IA: Invasive adenocarcinoma, pGGN: Pure ground glass nodules, mGGN: Mixed ground glass nodules, SN: Solid nodule. Figure S5. Overview of transcriptome data. A RNA-Seq passed reads sequenced by Illumina NoveSeq 6000 Nanopor [file 12967_2024_5485_MOESM1_ESM.zip › Suppl-figs/Figure S5.pdf]

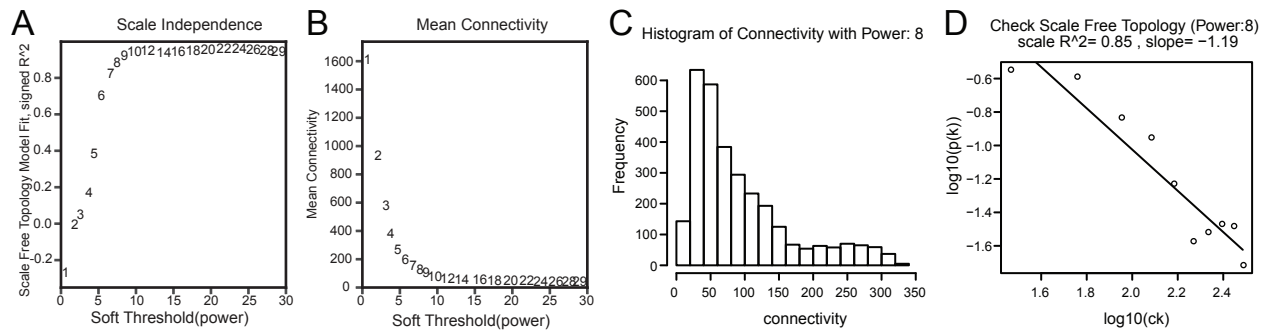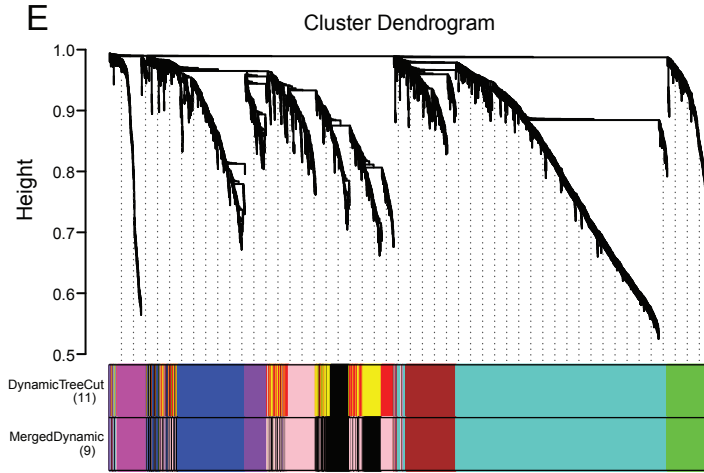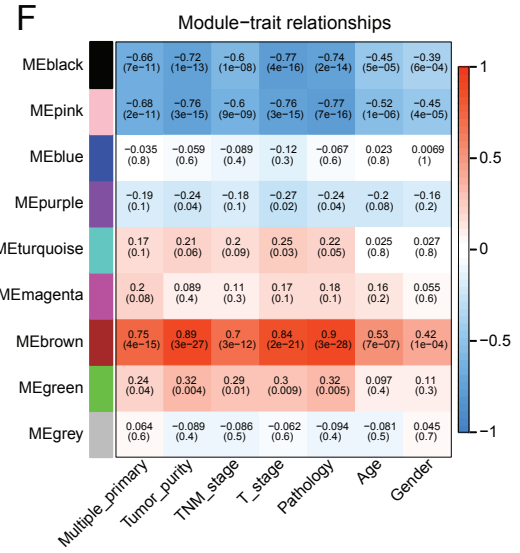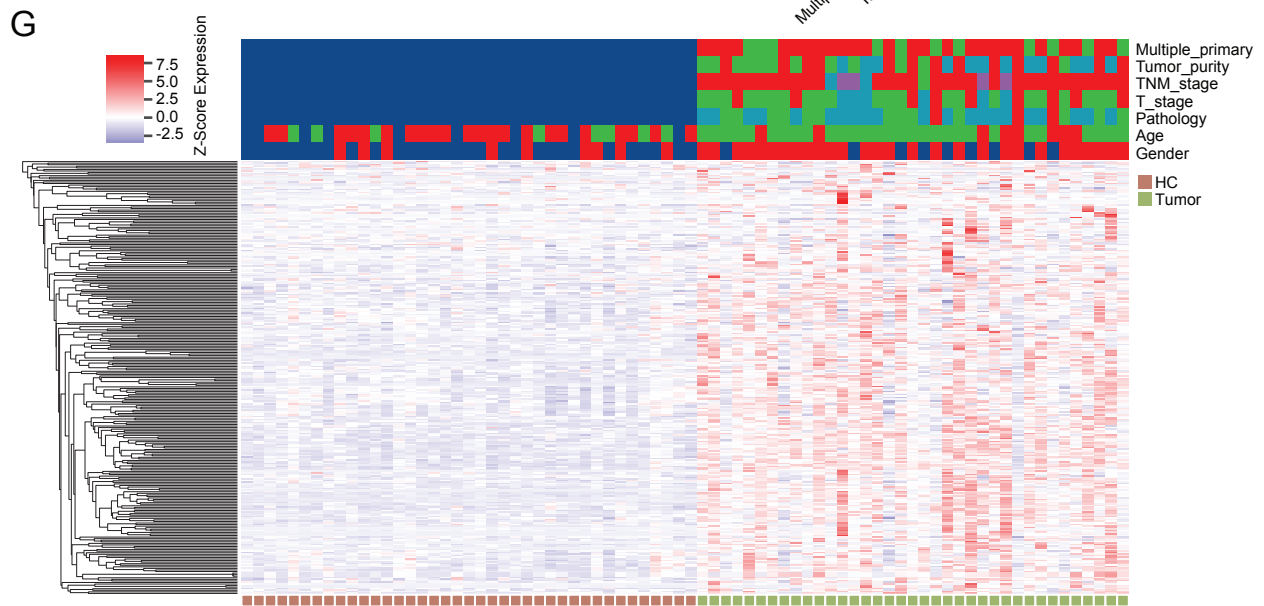

Supplement: Supplementary file 1 — Additional file 1: Figure S1. Quality control of metagenomic data. A Comparison of quality control passed reads after filtering in 129 samples. B Specaccum species accumulation curves of three groups. C Rarefaction curves showing observed species richness taken from the 129 samples. D Overall taxa distribution of the microbiome kingdom in three groups. Figure S2. Microbial compositions in the cohort. Microbial compositions of the patients with ESLUAD and HCs at the phylum (A), genus (B), and species (C) levels. The top 10/20 abundant microbial taxa are shown with different gradient colors. The microbial composition is arranged in order of the mostabundant taxonomic ranks. Figure S3. Representative microbes exhibiting significant alterations between patients with ESLUAD and HCs. *** p < 0.001 as determined by Kruskal–Wallis test. Figure S4. Correlation between intrapulmonary microbiota and clinical features. A, D Comparison of the alpha diversity (Chao1/Shannon/Simpson index) and beta diversity (Bray–Curtis distance) at the species level with tumor infiltration in patients with ES-LUAD. B, E Comparison of the alpha diversity (Chao1/Shannon/Simpson index) and beta diversity (Bray–Curtis distance) at the species level with solid component of tumor in patients with ES-LUAD. C, F Comparison of the alpha diversity (Chao1/Shannon/Simpson index) and beta diversity (Bray–Curtis distance) at the species level with multiple-primary nodules in patients with ES-LUAD. Box plots show median ± quartiles, and the whiskers extend from the hinge to the largest or smallest value no further than 1.5-fold of the interquartile range. ns: Not significant, p-value as determined by Wilcoxon rank-sum test. AIS: Adenocarcinoma in situ, MIA: Minimally invasive adenocarcinoma, IA: Invasive adenocarcinoma, pGGN: Pure ground glass nodules, mGGN: Mixed ground glass nodules, SN: Solid nodule. Figure S5. Overview of transcriptome data. A RNA-Seq passed reads sequenced by Illumina NoveSeq 6000 Nanopor [file 12967_2024_5485_MOESM1_ESM.zip › Suppl-figs/Figure S6.pdf]

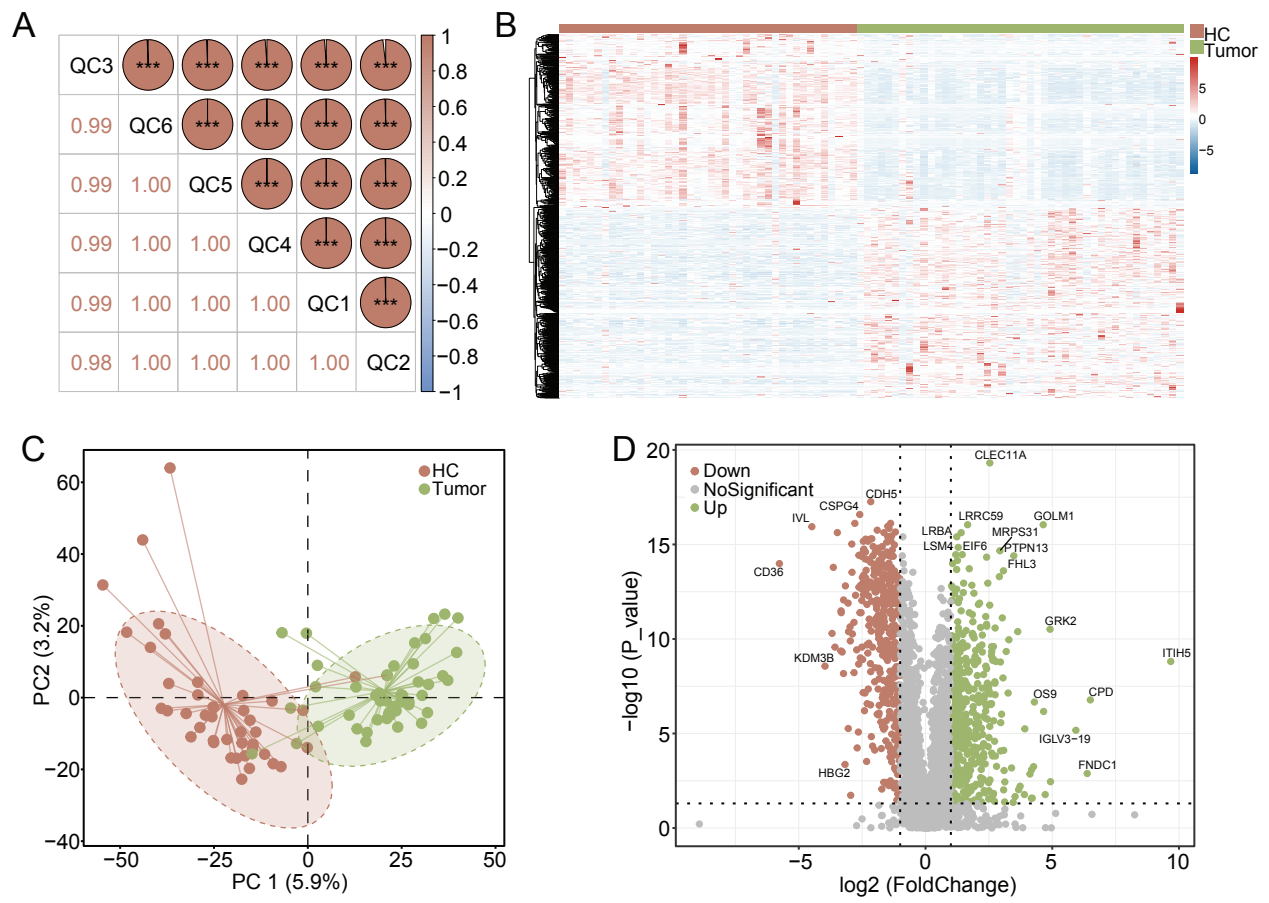

Supplement: Supplementary file 1 — Additional file 1: Figure S1. Quality control of metagenomic data. A Comparison of quality control passed reads after filtering in 129 samples. B Specaccum species accumulation curves of three groups. C Rarefaction curves showing observed species richness taken from the 129 samples. D Overall taxa distribution of the microbiome kingdom in three groups. Figure S2. Microbial compositions in the cohort. Microbial compositions of the patients with ESLUAD and HCs at the phylum (A), genus (B), and species (C) levels. The top 10/20 abundant microbial taxa are shown with different gradient colors. The microbial composition is arranged in order of the mostabundant taxonomic ranks. Figure S3. Representative microbes exhibiting significant alterations between patients with ESLUAD and HCs. *** p < 0.001 as determined by Kruskal–Wallis test. Figure S4. Correlation between intrapulmonary microbiota and clinical features. A, D Comparison of the alpha diversity (Chao1/Shannon/Simpson index) and beta diversity (Bray–Curtis distance) at the species level with tumor infiltration in patients with ES-LUAD. B, E Comparison of the alpha diversity (Chao1/Shannon/Simpson index) and beta diversity (Bray–Curtis distance) at the species level with solid component of tumor in patients with ES-LUAD. C, F Comparison of the alpha diversity (Chao1/Shannon/Simpson index) and beta diversity (Bray–Curtis distance) at the species level with multiple-primary nodules in patients with ES-LUAD. Box plots show median ± quartiles, and the whiskers extend from the hinge to the largest or smallest value no further than 1.5-fold of the interquartile range. ns: Not significant, p-value as determined by Wilcoxon rank-sum test. AIS: Adenocarcinoma in situ, MIA: Minimally invasive adenocarcinoma, IA: Invasive adenocarcinoma, pGGN: Pure ground glass nodules, mGGN: Mixed ground glass nodules, SN: Solid nodule. Figure S5. Overview of transcriptome data. A RNA-Seq passed reads sequenced by Illumina NoveSeq 6000 Nanopor [file 12967_2024_5485_MOESM1_ESM.zip › Suppl-figs/Figure S7.pdf]

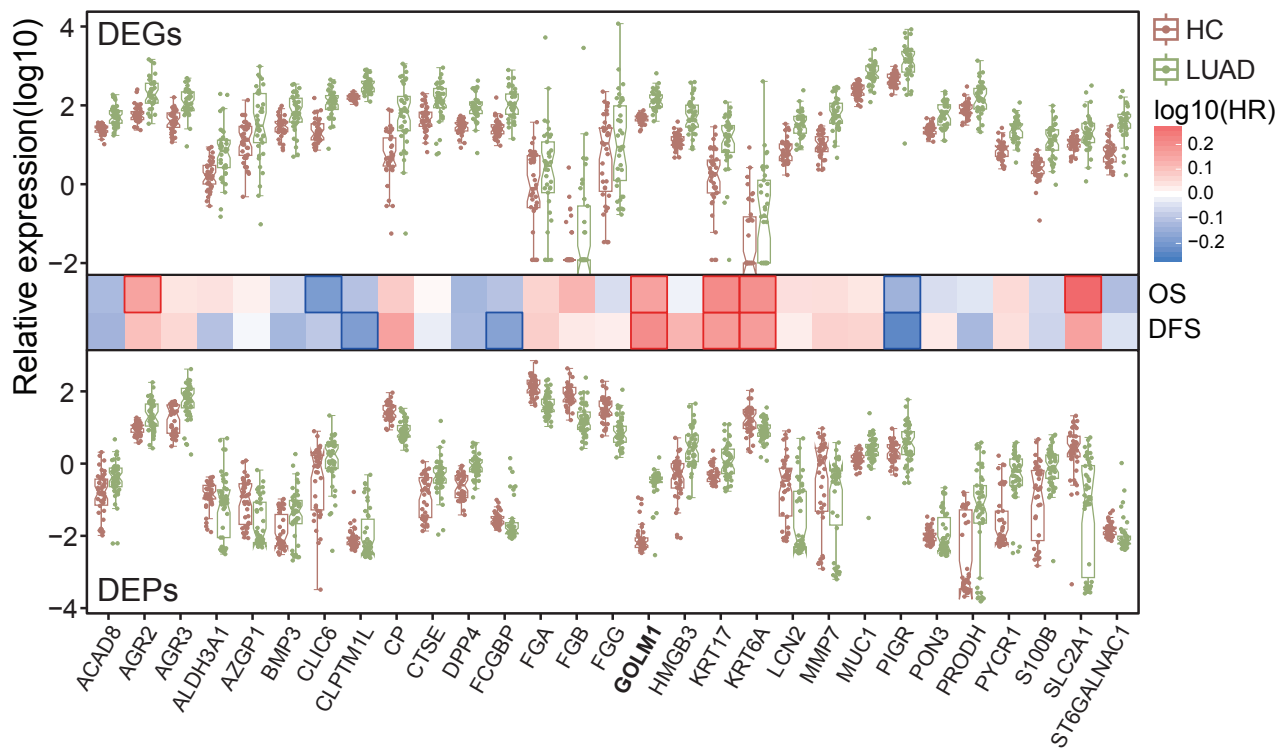

Supplement: Supplementary file 1 — Additional file 1: Figure S1. Quality control of metagenomic data. A Comparison of quality control passed reads after filtering in 129 samples. B Specaccum species accumulation curves of three groups. C Rarefaction curves showing observed species richness taken from the 129 samples. D Overall taxa distribution of the microbiome kingdom in three groups. Figure S2. Microbial compositions in the cohort. Microbial compositions of the patients with ESLUAD and HCs at the phylum (A), genus (B), and species (C) levels. The top 10/20 abundant microbial taxa are shown with different gradient colors. The microbial composition is arranged in order of the mostabundant taxonomic ranks. Figure S3. Representative microbes exhibiting significant alterations between patients with ESLUAD and HCs. *** p < 0.001 as determined by Kruskal–Wallis test. Figure S4. Correlation between intrapulmonary microbiota and clinical features. A, D Comparison of the alpha diversity (Chao1/Shannon/Simpson index) and beta diversity (Bray–Curtis distance) at the species level with tumor infiltration in patients with ES-LUAD. B, E Comparison of the alpha diversity (Chao1/Shannon/Simpson index) and beta diversity (Bray–Curtis distance) at the species level with solid component of tumor in patients with ES-LUAD. C, F Comparison of the alpha diversity (Chao1/Shannon/Simpson index) and beta diversity (Bray–Curtis distance) at the species level with multiple-primary nodules in patients with ES-LUAD. Box plots show median ± quartiles, and the whiskers extend from the hinge to the largest or smallest value no further than 1.5-fold of the interquartile range. ns: Not significant, p-value as determined by Wilcoxon rank-sum test. AIS: Adenocarcinoma in situ, MIA: Minimally invasive adenocarcinoma, IA: Invasive adenocarcinoma, pGGN: Pure ground glass nodules, mGGN: Mixed ground glass nodules, SN: Solid nodule. Figure S5. Overview of transcriptome data. A RNA-Seq passed reads sequenced by Illumina NoveSeq 6000 Nanopor [file 12967_2024_5485_MOESM1_ESM.zip › Suppl-figs/Figure S8.pdf]

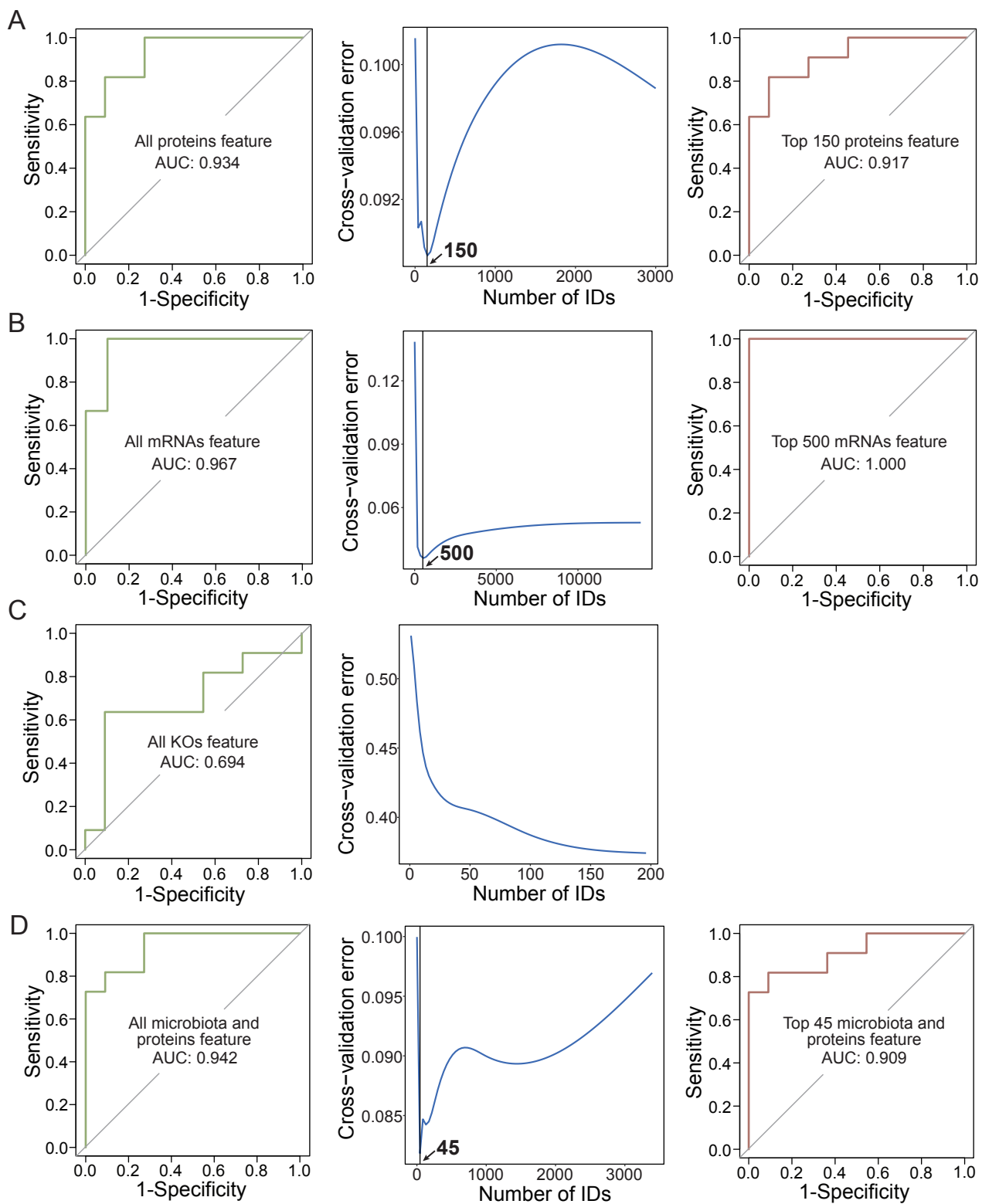

Supplement: Supplementary file 1 — Additional file 1: Figure S1. Quality control of metagenomic data. A Comparison of quality control passed reads after filtering in 129 samples. B Specaccum species accumulation curves of three groups. C Rarefaction curves showing observed species richness taken from the 129 samples. D Overall taxa distribution of the microbiome kingdom in three groups. Figure S2. Microbial compositions in the cohort. Microbial compositions of the patients with ESLUAD and HCs at the phylum (A), genus (B), and species (C) levels. The top 10/20 abundant microbial taxa are shown with different gradient colors. The microbial composition is arranged in order of the mostabundant taxonomic ranks. Figure S3. Representative microbes exhibiting significant alterations between patients with ESLUAD and HCs. *** p < 0.001 as determined by Kruskal–Wallis test. Figure S4. Correlation between intrapulmonary microbiota and clinical features. A, D Comparison of the alpha diversity (Chao1/Shannon/Simpson index) and beta diversity (Bray–Curtis distance) at the species level with tumor infiltration in patients with ES-LUAD. B, E Comparison of the alpha diversity (Chao1/Shannon/Simpson index) and beta diversity (Bray–Curtis distance) at the species level with solid component of tumor in patients with ES-LUAD. C, F Comparison of the alpha diversity (Chao1/Shannon/Simpson index) and beta diversity (Bray–Curtis distance) at the species level with multiple-primary nodules in patients with ES-LUAD. Box plots show median ± quartiles, and the whiskers extend from the hinge to the largest or smallest value no further than 1.5-fold of the interquartile range. ns: Not significant, p-value as determined by Wilcoxon rank-sum test. AIS: Adenocarcinoma in situ, MIA: Minimally invasive adenocarcinoma, IA: Invasive adenocarcinoma, pGGN: Pure ground glass nodules, mGGN: Mixed ground glass nodules, SN: Solid nodule. Figure S5. Overview of transcriptome data. A RNA-Seq passed reads sequenced by Illumina NoveSeq 6000 Nanopor [file 12967_2024_5485_MOESM1_ESM.zip › Suppl-figs/Figure S9.pdf]
